# Phosphorus deficiency alleviates iron limitation in *Synechocystis* cyanobacteria through direct PhoB-mediated gene regulation

**DOI:** 10.1038/s41467-024-48847-4

**Published:** 2024-05-24

**Authors:** Guo-Wei Qiu, Wen-Can Zheng, Hao-Ming Yang, Yu-Ying Wang, Xing Qi, Da Huang, Guo-Zheng Dai, Huazhong Shi, Neil M. Price, Bao-Sheng Qiu

**Affiliations:** 1 School of Life Sciences, Hubei Key Laboratory of Genetic Regulation and Integrative Biology, Central China Normal University, Wuhan, Hubei 430079 China; 2 Department of Chemistry and Biochemistry, Texas Tech University, Lubbock, TX 79409 USA; 3 Department of Biology, McGill University, 1205 Docteur Penfield, Montreal, Québec H3A 1B1 Canada

**Keywords:** Water microbiology, Microbial ecology, Bacterial physiology

## Abstract

Iron and phosphorus are essential nutrients that exist at low concentrations in surface waters and may be co-limiting resources for phytoplankton growth. Here, we show that phosphorus deficiency increases the growth of iron-limited cyanobacteria (*Synechocystis* sp. PCC 6803) through a PhoB-mediated regulatory network. We find that PhoB, in addition to its well-recognized role in controlling phosphate homeostasis, also regulates key metabolic processes crucial for iron-limited cyanobacteria, including ROS detoxification and iron uptake. Transcript abundances of PhoB-targeted genes are enriched in samples from phosphorus-depleted seawater, and a conserved PhoB-binding site is widely present in the promoters of the target genes, suggesting that the PhoB-mediated regulation may be highly conserved. Our findings provide molecular insights into the responses of cyanobacteria to simultaneous iron/phosphorus nutrient limitation.

## Abstract (web summary)

Iron and phosphorus exist at low concentrations in surface waters and may be co-limiting resources for phytoplankton growth. Here, the authors show that phosphorus deficiency increases the growth of iron-limited cyanobacteria through a PhoB-mediated regulatory network.

## Introduction

Nutrient availability constrains the rate of biological production and the structure of ecosystem communities on a global scale^1,2^. As essential nutrients required by all living organisms, iron (Fe) and phosphorus (P) participate in a series of metabolic processes, and their chemical roles are deeply interconnected^3–5^. While the critical role of Fe and P in controlling primary productivity has long been recognized^6–8^, recent studies demonstrate that limitation by Fe and P could occur simultaneously^9,10^. Such Fe/P co-limitation has been observed in large areas of the low-latitude oceans as well as freshwater ecosystems^9–12^. It might also be prevalent in terrestrial environments due to the low bioavailability of both elements in alkaline and calcareous soils, which represent one-third of the world’s cultivated lands^13,14^. However, despite the apparent issue of Fe/P co-limitation, we know very little about the adaptive strategies employed by the organisms living in co-limited environments, especially at the level of gene regulation.

Recent studies suggest that marine cyanobacteria experiencing Fe/P co-limitation may have established specific responses, as several co-limited diazotrophic cyanobacteria can maintain faster rates of growth than when limited by either nutrient alone^15–17^. A general mechanism that facilitates this enhanced fitness could be cell size reduction, which is observed in co-limited *Crocosphaera* and *Trichodesmium*^16,17^. Decreasing cell size under Fe/P co-limited conditions enables these large-sized cyanobacteria to alleviate their high demand on cellular elements and optimize their surface area-to-volume ratio for nutrient uptake^18,19^. However, this strategy may not be appropriate for small-sized cyanobacteria. It has been demonstrated that phytoplankton achieve the maximal growth rate at intermediate cell sizes^20,21^. For cyanobacteria with a radius smaller than 0.9 µm, further compression of their cell size will result in an overallocation of the resources to non-scalable components, e.g., genome and membranes, which could decrease resource availability for other essential activities^22^. Their growth rate is thus reduced. Moreover, small cells are more vulnerable to grazing^19^. Such reduced growth rates and increased grazing mortality may result in a severe decline of small-sized cyanobacterial communities.

It should be noted that small-sized cyanobacteria, including *Prochlorococcus* and *Synechococcus*, are the most abundant and dominant primary producers worldwide^23^. Their ability to respond to Fe/P co-limitation could have significant consequences for carbon sequestration and biogeochemical cycling. Interesting questions include whether cyanobacteria from the intermediate or small size range also exhibit increased fitness under Fe/P co-limitation, and if so, by what mechanism.

Recent study in land plants reveals that P deficiency prevents the accumulation of Fe deficiency-induced reactive oxygen species (ROS) by inducing ascorbate biosynthesis^24^. However, the in vivo ascorbate concentration of cyanobacteria is 250 times lower than that in chloroplasts^25^, and the gene encoding ascorbate peroxidase is absent from cyanobacteria^26^, suggesting a limited role of ascorbate in cyanobacterial ROS detoxification. Whether P deficiency also affects ROS cycling in Fe-limited cyanobacteria remains unanswered. Moreover, although the expression of cyanobacterial Fe uptake genes is typically regulated by the Fur repressor in a cytoplasmic Fe level-dependent manner^27^, the role of other environmental factors, such as P-limitation, in the regulation of Fe uptake genes needs to be determined.

Here, we investigated the adaptive strategy employed by Fe/P co-limited cyanobacteria in a genetically operable intermediate-sized strain, *Synechocystis* sp. PCC 6803 (hereafter *Synechocystis* 6803). Our results indicate that P deficiency increases the growth of Fe-limited cyanobacteria through a PhoB-mediated regulatory network. The presence of a direct regulation between Fe and P homeostasis via PhoB could enable cyanobacteria to allocate better their metabolic processes under nutrient-fluctuating environments where deficiency or enrichment of these two elements frequently occurs simultaneously.

## Results

### P deficiency alleviates the growth defect of Fe-limited cyanobacteria

**Fig. 1:**
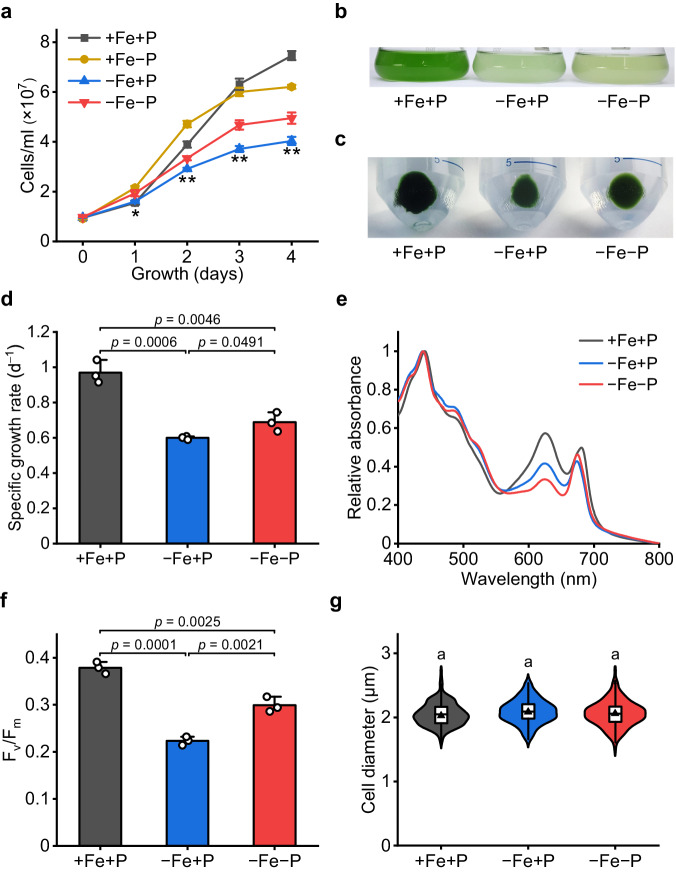
Phosphorus starvation enhances the growth of Fe-limited cyanobacteria. **a** The growth curve of *Synechocystis* under different iron and phosphorus availabilities. Values are mean ± SD of three independent biological replicates. Asterisks indicate a significant difference between −Fe+P and −Fe−P using the two-sided Student’s *t-*test. *P < 0.05, **P < 0.01, with exact *P-*values provided as source data. **b**–**c** Image of the culture and biomass of *Synechocystis* after 4 days of growth. The biomass shown in (**c**) was the pellets obtained from 100 mL of cells after centrifugation. The experiment was repeated with similar results three times. **d** Growth rates of *Synechocystis* grown under +Fe+P, −Fe+P, and −Fe−P conditions. Values are mean ± SD of three independent biological replicates. **e** Room temperature absorption spectra of *Synechocystis* grown under +Fe+P, −Fe+P, and −Fe−P conditions for 4 days. Data shown are from representative sample of three independent biological replicates. **f** Changes in Fv/Fm values of *Synechocystis* after 4 days of growth under +Fe+P, −Fe+P, and −Fe−P conditions. Values are mean ± SD of three independent biological replicates. **g** The violin plot showing the distribution of the cell diameter data points, while the embedded line and triangle in the boxplot mark the mean and median cell diameter, respectively. The upper and lower edges of the box indicate the first and third quartiles, and the whiskers extend to 1.5 × the interquartile range beyond the first and third quartiles. Data shown are measured from three independent biological replicates with the total number of at least 150 cells. Letters above bars indicate statistical significance (*P* < 0.05) calculated by one-way ANOVA across all treatments. For **d** and **f** line segments and corresponding *P*-values represent statistical significance calculated by two-sided Student’s *t*-test. Source data are provided as a Source Data file.

The physiological response of *Synechocystis* 6803 to Fe/P co-limitation was examined under different availabilities of Fe and P. As expected, Fe starvation alone (−Fe+P) strongly impeded the growth of cyanobacteria (Fig. 1a–d). However, this effect was clearly alleviated by P deficiency. Fe/P co-limited *Synechocystis* grew 15% faster than the cells experiencing Fe starvation only (Fig. 1d). After culture for 4 days, more biomass accumulated under −Fe−P than −Fe+P conditions (Fig. 1c). It should be noted that both Fe limitation (*isiA*) and P limitation (*pstS*, *pstS2*) biomarker genes were strongly induced in the Fe/P co-limited cells (−Fe−P, Supplementary Fig. 1). The blue shift of the red absorption peak of chlorophyll observed in room temperature absorption spectra of co-limited culture was also consistent with the induction of IsiA (Fig. 1e).

Photosynthetic performance of cells was significantly higher in the −Fe−P than in −Fe+P treatment (Fig. 1f). However, this enhanced Fv/Fm was not caused by an increase in content of the two photosystems. Immunoblotting with specific antibodies revealed that thylakoid membranes of cells in the −Fe−P treatment contained a similar amount of PSI complexes as cells in the −Fe+P treatment, whereas the protein level of the PSII complexes was lower in the −Fe−P treated samples (Supplementary Fig. 2). Overall, these results consistently showed that P deficiency alleviated the growth defect of Fe-limited *Synechocystis*. Both microscopy (Fig. 1g) and FACS analyses (Supplementary Fig. 3) indicated no significant variation in cell size under any of the experimental conditions, implying that the underlying mechanism was cell-size independent.

### The expression of *sod* is up-regulated under Fe/P co-limited conditions

**Fig. 2:**
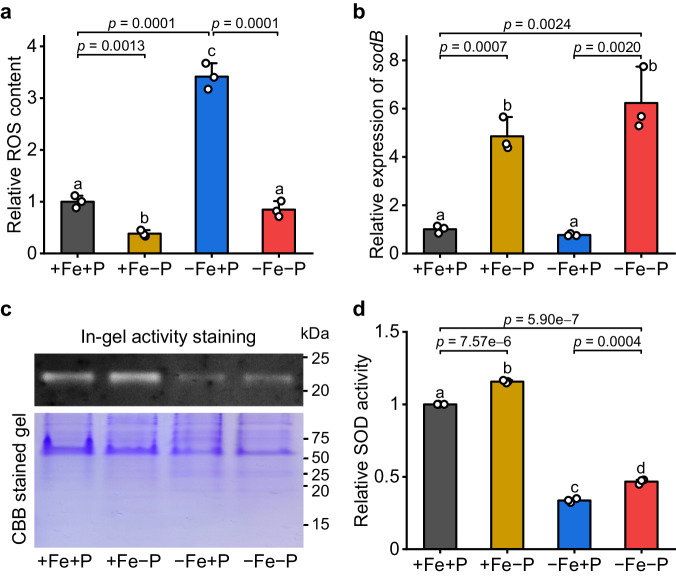
*sodB* responds positively to phosphorus starvation. **a** The intracellular ROS accumulation of *Synechocystis* after 4 days of growth under different iron and phosphorus availabilities. Values are mean ± SD of three independent biological replicates, and the results were normalized to the ROS level of +Fe+P treatment. **b** Relative mRNA abundance of *sodB* in *Synechocystis* after 4 days of growth under +Fe+P, +Fe−P, −Fe+P, and −Fe−P conditions. Data shown are mean ± SD of three independent biological replicates, and the results were normalized to the transcript levels measured under +Fe+P conditions. **c** SOD activity of *Synechocystis* determined by in-gel activity staining. Samples were loaded on an equal total protein basis. Similar results were obtained from three independent biological replicates. **d** The SOD activity detected from (**c**) was quantified with ImageJ software. Data shown are mean ± SD of three independent biological replicates and values are given relative to SOD activities of +Fe+P samples. Letters above bars indicate statistical significance (*P* < 0.05) calculated by one-way ANOVA across all treatments. Line segments and corresponding *P*-values represent statistical significance calculated by two-sided Student’s *t*-test. Source data are provided as a Source Data file.

Previous work showed that massive accumulation of ROS under Fe-limited conditions could have a severe effect on photosynthetic activity of cyanobacteria^28^. We thus quantified the total ROS levels and found that the ROS content of −Fe−P cells was reduced by 75% compared with −Fe+P cells (Fig. 2a), indicating enhanced ROS scavenging activities were induced by low P under Fe deficiency. qRT-PCR analysis showed that several genes involved in ROS scavenging were more highly expressed under −Fe−P than −Fe+P conditions (Supplementary Fig. 4). Among the differentially expressed genes, the iron-containing superoxide dismutase (SOD) coding gene, *sodB*, was the most up-regulated. Further analysis revealed that the expression of *sodB* was significantly up-regulated in the +Fe−P and −Fe−P samples (Fig. 2b), indicating P deficiency enhanced *sodB* expression. This pattern was further supported by SOD assays using in-gel activity staining (Fig. 2c, d). SOD activity of *Synechocystis* was sharply reduced by Fe starvation and increased under P deficiency: SOD activity was 39% higher in −Fe−P than in −Fe+P grown cells (Fig. 2d). The enhanced SOD activity may account for the decreased ROS accumulation in Fe/P co-limited *Synechocystis* (Fig. 2a).

### Phosphorus availability modulates *sod* expression in the world’s oceans

**Fig. 3:**
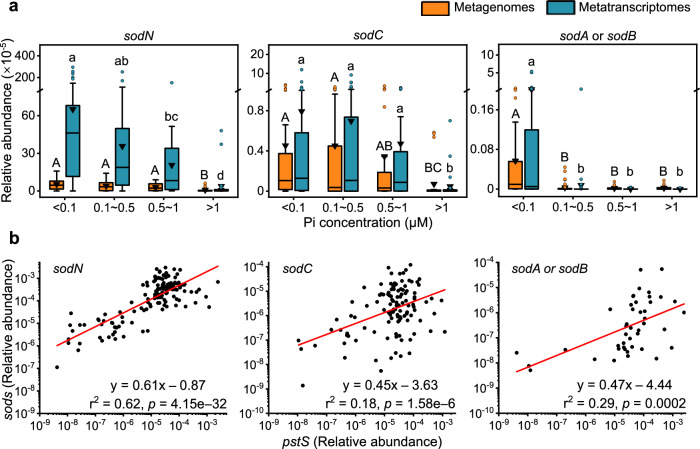
Distribution of *sods* amongst marine ecosystems. **a** Whisker box plots showing gene abundance of different *sod* isoforms of cyanobacteria in metagenomes (orange, *n*_*mg*_ = 139) and metatranscriptomes (dark cyan, *n*_mt_ = 162) generated from TARA oceans global marine survey. Data are presented by Pi (<0.1 µM, *n*_*mg*_ = 39, *n*_*mt*_ = 40; 0.1 ~ 0.5 µM, *n*_*mg*_ = 49, *n*_*mt*_ = 60; 0.5 ~ 1.0 µM, *n*_*mg*_ = 26, *n*_*mt*_ = 34; >1.0 µM, *n*_*mg*_ = 25, *n*_*mt*_ = 28) concentrations. The abundance of each *sod* was calculated by dividing the sum of the abundances of their homologs by the sum of total gene abundance from all reads in the sample. The whiskers extend to 1.5 × the interquartile range beyond the first and third quartiles (boxes). Exclusive median (line), average (triangle), and atypical values (circles) are also indicated. Different letters above the bars represent statistical significance (*P* < 0.05) calculated by Kruskal-Wallis between treatments. **b** Plots of the relative *sods* abundance in metatranscriptomes vs. the relative level of *pstS* transcripts. The data were plotted on a logarithmic scale. The red lines indicate linear regressions of the data points, and the significance of each regression is shown in each panel. Source data are provided as a Source Data file.

The laboratory experiments demonstrated that P deficiency induced *sodB* expression under Fe sufficient and deficient conditions, so we examined if *sod* genes and transcripts of marine cyanobacteria also varied with nutrient availability. SOD exists in four isoforms, each with different metal cofactors^29,30^. Only *sodB* utilizes Fe as its cofactor, whereas other *sods* use either manganese (*sodA*), nickel (*sodN*), or a combination of copper and zinc (*sodC*) as co-factors. The distribution pattern of *sods* in the global marine TARA metagenome datasets^31^ showed that *sods* are widely present in cyanobacteria that thrive under different levels of phosphate (Pi) bioavailability (Fig. 3a). Notably, *Prochlorococcus* and *Synechococcus* represent the vast majority of the hits of the *sods*, and the large reduction in *sods* abundance in relatively high-Pi regions (>1 µM) is consistent with the low abundance of both genera in polar oceans^23^.

Metatranscriptomic analysis revealed that the expression of different *sod* isoforms was enriched in low-Pi regions (<0.5 µM, Fig. 3a and Supplementary Fig. 5). Considering the complexity of marine phosphorus cycle, we further examined the relative levels of *sods* transcripts versus that of the P limitation biomarker, *pstS*^10,17^. A positive relationship between their abundances was observed (Fig. 3b). These data consistently show that *sods* expression was enhanced by P deficiency in the ocean and could be a common response of cyanobacteria.

### Phosphorus deficiency enhances O_2_^−^ resistance of Fe-limited cyanobacteria

**Fig. 4:**
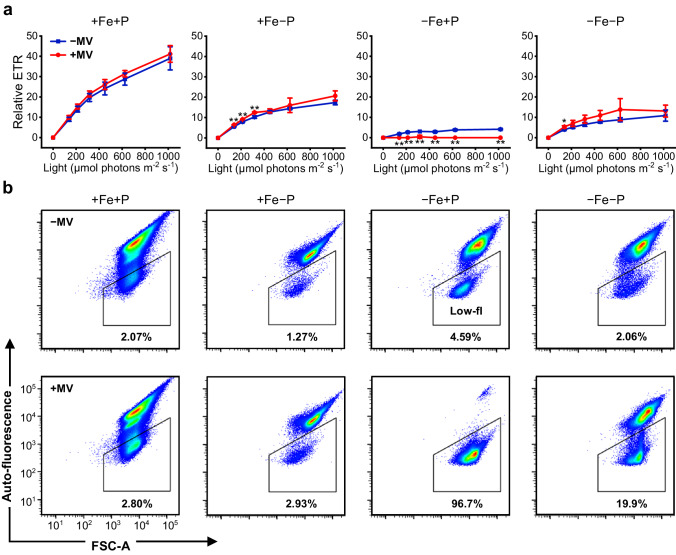
Phosphorus starvation enhances the resistance of Fe-limited cyanobacteria to O_2_^−^ stress. **a** The photosynthetic electron transport rate of *Synechocystis* grown in the presence (+MV) and absence (−MV) of 0.3 µM methyl viologen measured by PAM fluorometry. Values are mean ± SD of three independent biological replicates. Asterisks indicate a significant difference between two treatments using the two-sided Student’s *t*-test. *P < 0.05; **P < 0.01. **b** Representative gating of low auto-fluorescence cells. The change of auto-fluorescence intensity of *Synechocystis* cells was analyzed by flow cytometry with an excitation wavelength of 488 nm and an emission wavelength of 700 ± 54 nm. Data shown are from representative sample of three independent biological replicates. Low-fl, low auto-fluorescence; FSC, forward scatter. Source data are provided as a Source Data file.

To explore the influence of *sod* induction on the growth of Fe-limited cyanobacteria, an O_2_^−^-tolerance experiment was performed in the presence and absence of methyl viologen (MV), respectively. MV efficiently accepts electrons from PSI and reduces oxygen to O_2_^−^, allowing us to assess the resistance of cyanobacteria to O_2_^−^^32^. As shown in Fig. 4, Fe-limited cyanobacteria were highly sensitive to O_2_^−^ stress. The photosynthetic electron transport rate (ETR) of −Fe+P samples was significantly decreased in the presence of the O_2_^−^ inducer ( +MV) compared to the treatment without MV (−MV, Fig. 4a). After incubation with MV for 8 days, the ETR of −Fe+P samples was nearly undetectable. In contrast, when P deficiency co-occurred with −Fe, the resistance of Fe-limited cells (−Fe−P) to O_2_^−^ was clearly enhanced (Fig. 4a). Similar results were also observed at the single-cell level (Fig. 4b and Supplementary Fig. 6). At the end of the treatment ( + MV, 10th day), up to 97% of −Fe+P cells lost chlorophyll auto-fluorescence and became chlorotic (low-fl). However, only 20% of the cells were chlorotic in the −Fe−P sample (Fig. 4b and Supplementary Fig. 6). These results demonstrated that P deficiency can strongly enhance the O_2_^−^ tolerance of Fe-limited cyanobacteria.

### The up-regulation of *sodB* is mediated by the transcription factor PhoB

**Fig. 5:**
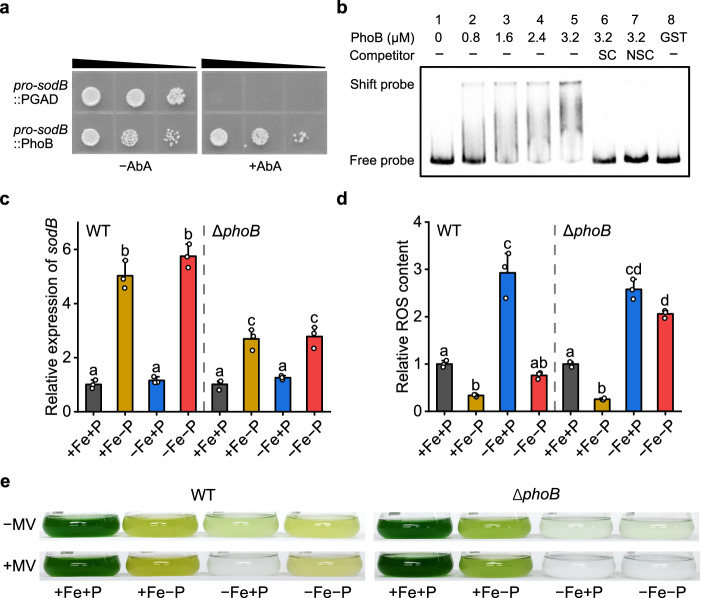
PhoB regulates the expression of *sodB.* **a** Verification of the interaction between the *cis*-acting element *pro*-*sodB* and the transcription factor PhoB by Y1H assay. **b** Interaction analysis between *pro*-*sodB* and PhoB by EMSA. The concentration of PhoB added into each mixture is shown. Unlabeled DNA (SC) and poly (dI-dC) (NSC) were added to examine the specificity of the binding reactions. Similar results were obtained from three independent experiments. **c** The relative transcript levels of *sodB* in the WT strain and *phoB* mutant after 4 days of growth under different Fe and P availabilities. Values are mean ± SD of three independent biological replicates, and the results were normalized to the transcript levels measured under +Fe+P conditions. **d** Intracellular ROS content of WT and the *phoB* mutant after 4 days of growth under +Fe+P, +Fe−P, −Fe+P, and −Fe−P conditions. Data shown are mean ± SD of three independent biological replicates, and the results were normalized to the ROS level of +Fe+P treatment. **e** Representative images of each strain grown in the absence and presence of 0.3 µM MV. The experiment was repeated with similar results three times. For **c** and **d** letters above bars indicate statistical significance (*P* < 0.05) calculated by one-way ANOVA across all treatments. Source data are provided as a Source Data file.

To decode the signaling pathways that induce *sodB* expression under Fe/P co-limitation, the transcription factor library of *Synechocystis* was screened using a yeast one-hybrid (Y1H) system with the *sodB* promoter as a bait. Interestingly, an OmpR family transcription factor, PhoB, was identified as a putative regulator (Fig. 5a). Bioinformatic analysis indicated a putative PhoB binding site presents in the promoter region of *sodB* (Supplementary Fig. 7a). The interaction between PhoB and the *sodB* promoter was further verified by an electrophoretic mobility shift assay (EMSA, Fig. 5b). PhoB is a transcription activator known to control the expression of phosphorus assimilating genes under low ambient Pi concentrations^33–35^, however to our knowledge, its function in activating SOD gene expression has not been established previously.

To assess the regulatory role of PhoB in *sodB* gene expression, the *phoB* gene was knocked out by insertional inactivation (Supplementary Fig. 8a). As shown in Fig. 5c, the induction of *sodB* was significantly reduced in the *phoB* mutant. The transcript level of *sodB* increased 5.8-fold in the wild-type (WT) strain under −Fe−P conditions, while only a 2.8-fold increase was observed in the *phoB* mutant. The residual *sodB* induction in the *phoB* mutant could be ascribed to the transcription factor RpaB, a known regulator in oxidative stress response^36^. In fact, we identified that RpaB interacted with the *sodB* promoter in our Y1H library screening assay (Supplementary Fig. 9). Further analysis revealed that the intracellular ROS content in co-limited cells was only 26% of that under −Fe+P in the WT, while in the *phoB* mutant, this value was 80% (Fig. 5d). The decrease in *sodB* induction significantly reduced MV tolerance of the co-limited cyanobacteria (Fig. 5e). The O_2_^−^ tolerance assay showed that, when *phoB* was knocked out, P deficiency no longer enhanced the O_2_^−^ resistance of Fe-limited cyanobacteria, and the −Fe−P cells showed a response resembling the −Fe+P cells (Fig. 5e and Supplementary Fig. 10). We overexpressed *sodB* in *Synechocystis* (strain SodB-OE) to explore the role of SodB in ROS homeostasis. The SodB-OE strain showed a strong reduction in ROS content under Fe-limited conditions (Supplementary Fig. 11b), but had a significantly reduced growth rate (Supplementary Fig. 11c–d). Considering that SodB is Fe-dependent, high expression of this metalloenzyme likely increased the demand for cellular Fe, resulting in more severe Fe deficiency and slower growth. Altogether, these results consistently suggest that PhoB mediated *sodB* expression is essential for the maintenance of ROS homeostasis under −Fe−P conditions.

### PhoB regulates the expression of the Fe-selective porin

**Fig. 6:**
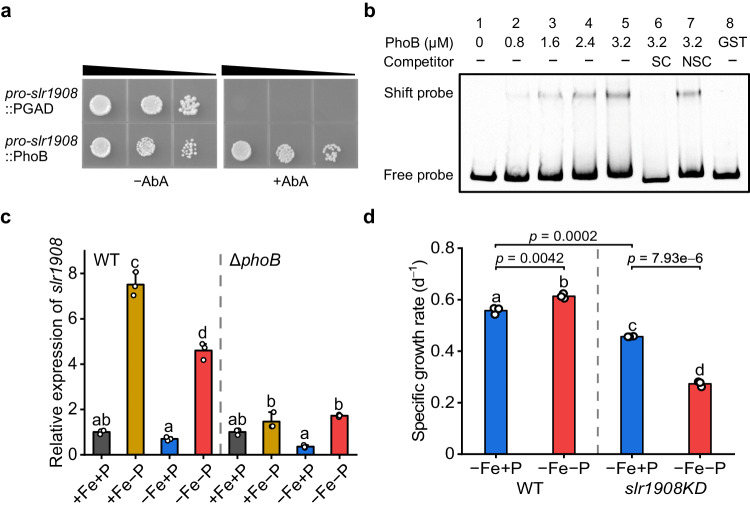
PhoB regulates the expression of *slr1908.* **a** Verification of the interaction between the *cis*-acting element *pro*-*slr1908* and the transcription factor PhoB by Y1H assay. **b** Interaction analysis between *pro*-*slr1908* and PhoB by EMSA. The concentration of PhoB added into each mixture is shown. Unlabeled DNA (SC) and poly (dI-dC) (NSC) were added to examine the specificity of the binding reactions. Similar results were obtained from three independent experiments. **c** qRT-PCR analysis of *slr1908* in WT strains and *phoB* mutants after 4 days of growth under different Fe and P availabilities. Values are mean ± SD of three independent biological replicates, and the results were normalized to the transcript levels measured under +Fe+P conditions. **d** Growth rates of the WT and *slr1908*KD strains grown under −Fe+P and −Fe−P conditions. Values are mean ± SD of three independent biological replicates. Line segments and corresponding *P*-values represent statistical significance calculated by two-sided Student’s *t*-test. For **c** and **d** letters above bars indicate statistical significance (*P* < 0.05) calculated by one-way ANOVA across all treatments. Source data are provided as a Source Data file.

When screening the potential transcription factors for our previously identified iron-selective porin^37^, *slr1908*, we found that the expression of this Fe uptake gene was also regulated by PhoB (Fig. 6). The specific interaction between PhoB and the *slr1908* promoter was further verified by both Y1H (Fig. 6a) and EMSA assays (Fig. 6b). Gene expression analysis revealed that, in the WT, the transcript level of *slr1908* was strongly induced by P deficiency, being up-regulated by 7.5- and 4.6-fold under +Fe−P and −Fe−P conditions, respectively (Fig. 6c). This P deficiency-induced Slr1908 expression was also confirmed at the protein level (Supplementary Fig. 12). However, the induced expression of *slr1908* was substantially reduced in the *phoB* mutant (Fig. 6c).

Because the complete knockout mutant of *slr1908* is not available in our *Synechocystis* sub-strains^37^, the physiological role of *slr1908* under Fe/P co-limitation was first assessed in a knockdown (KD) mutant. Compared to the WT, growth of the *slr1908*KD strain was significantly reduced under −Fe+P conditions (Fig. 6d and Supplementary Fig. 13a), consistent with the gene’s essential role in Fe uptake^37^. Surprisingly, the growth of *slr1908*KD strain was not enhanced under Fe/P co-limitation compared to the Fe-limited conditions. The growth rate and cell density of the co-limited *slr1908*KD strain was even lower than that under −Fe+P conditions (Fig. 6d and Supplementary Fig. 13a). Moreover, overexpression of *slr1908* significantly enhanced the growth rate of Fe-limited cyanobacteria (Supplementary Fig. 11d), suggesting a critical role of *slr1908* induction in acclimation to Fe-limitation. Given the important role of PhoB in low-P response^33^, our results demonstrate that PhoB also facilitates Fe uptake and plays a role in cross-talk between Fe and P homeostasis.

### PhoB also regulates genes involved in chemotaxis and exotoxin secretion

**Fig. 7:**
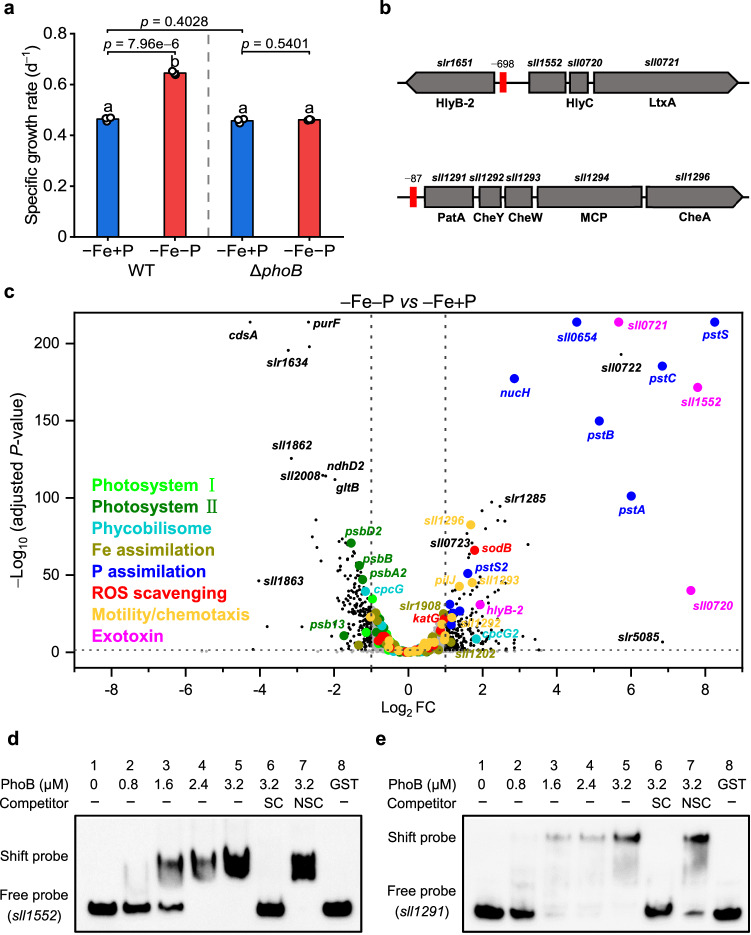
PhoB regulates chemotaxis and exotoxin related genes. **a** Growth rates of the WT and *phoB* mutants grown under −Fe+P and −Fe−P conditions. Values are mean ± SD of three independent biological replicates. Letters above bars indicate statistical significance (*P* < 0.05) calculated by one-way ANOVA across all treatments. Line segments and corresponding *P*-values represent statistical significance calculated by two-sided Student’s *t*-test. **b** The schematic representation of the organization of *sll1552* and *sll1291* gene clusters. The location of the putative PhoB binding sites were labeled by red. **c** Global expression analysis of genes in response to −Fe−P relative to −Fe+P. Differential expression testing was carried out using DEseq2 package, and two-tailed *P*-values were adjusted for multiple testing using the BH method. Broken lines indicate the adjusted *P*-value threshold of 0.05 and FC thresholds of 1 and −1. Functional groups are color coded and genes without significant differences in expression are shown in light gray. Detailed numeric values are presented in Supplementary Data 2. **d** and **e** Interaction analysis between *pro*-*sll1552*, *pro*-*sll1291* and PhoB by EMSA. The concentration of PhoB added into each mixture is shown. Unlabeled DNA (SC) and poly (dI-dC) (NSC) were added to examine the specificity of the binding reactions. Similar results were obtained from three independent experiments. Source data are provided as a Source Data file.

When *phoB* was inactivated, the positive effect of P deficiency on Fe-limited growth was greatly diminished (Fig. 7a and Supplementary Fig. 13b), confirming the importance of PhoB-mediated transcriptional regulation in the Fe/P co-limitation response. A phylogenetic footprinting technique was applied to the promoter regions of experimentally verified PhoB target genes to identify the PhoB-mediated genome-wide gene regulation. A conserved motif containing three tandem direct repeats was identified (Supplementary Fig. 7b). This motif was consistent with the previously reported *pho* box consensus sequence^38^, and the core sequence of the repeats (TTAA) was highly conserved (Supplementary Fig. 7c). EMSA assays demonstrated that mutations in any of these three TTAA sequences remarkably reduced or abolished the DNA-binding ability of PhoB (Supplementary Fig. 7d), indicating that the three tandem repeats are vital for recognition by PhoB. Based on the sequence of the core motif, 211 promoters were identified to contain a potential PhoB binding site. Of these, 92 potential target genes were up-regulated by P deficiency (FC ≥ 1.5, *P*-value ≤ 0.05) under either Fe sufficient or deficient conditions, consistent with the expression model of the PhoB targets (Supplementary Data 1). The results of this computational analysis were further verified by chromatin immunoprecipitation sequencing (ChIP-seq), and 60 of these 92 genes were enriched in our ChIP-seq results (Supplementary Data 1 and Supplementary Fig. 14).

In addition to its well-recognized role in controlling phosphate assimilation, newly identified PhoB targets included genes related to stress response, photosynthesis and tetrapyrrole metabolism (Supplementary Data 1). Amongst them was *sll1552*, a gene located upstream of the type I secretion system coding gene *slr1651* in the reverse direction, which may form an exotoxin biosynthesis cluster with the downstream acyltransferase gene *sll0720* and RTX (repeats in toxin) protein gene *sll0721* (Fig. 7b). All genes in this cluster were strongly induced by P deficiency as shown in our transcriptomic data (Fig. 7c) and qRT-PCR analysis (Supplementary Fig. 15a–c). Their induction in the *phoB* mutant was significantly reduced, consistent with the expression pattern of the Pho regulon^33^. The interaction between PhoB and their promoters was further verified by EMSA assay (Fig. 7d). RTX proteins can form pores in cytoplasmic membranes, leading to uncontrolled efflux of ions or nutrients from the host cells^39^. Previous research in *Actinobacillus* revealed that the production of RTX toxin is specifically induced by Fe deficiency^40^, indicating a potential role of this family in low Fe response. Moreover, several genes involved in type IV pilus biogenesis and chemotaxis were also identified as putative targets of PhoB (Supplementary Data 1). Regulation of the *sll1291* cluster by PhoB was further supported by our experimental results (Fig. 7e and Supplementary Fig. 15d–f). The up-regulation of genes involved in chemotaxis may provide a considerable competitive advantage to Fe-limited cyanobacteria to forage nutrient hotspots within patchy environments.

## Discussion

Recent studies have shown that cyanobacteria experiencing Fe/P co-limitation exhibit faster growth than those limited by a single nutrient^15–17^, but the regulatory mechanism responsible for such a response is poorly understood. Here, we present multiple lines of evidence that collectively support the critical role of a PhoB-mediated regulatory network in Fe/P co-limitation acclimation. PhoB is a conserved transcription factor of a two-component system with the sensor kinase PhoR. Under P-limited conditions, PhoR autophosphorylation leads to the phosphorylation of PhoB, triggering PhoB dimerization and DNA binding activity^35^. Although PhoB is typically considered to respond to phosphorus deficiency^33^, our results suggest that it may enhance the fitness of Fe/P co-limited cyanobacteria by maintaining intracellular ROS homeostasis and facilitating Fe uptake.

Under P deficiency, the NADPH/NADP^+^ ratio in photosynthetic organisms increases significantly, severely inhibiting linear electron flow^41^. Once the electron transport to ferredoxin-NADP^+^ oxidoreductase is blocked, oxygen may serve as an alternative electron acceptor, running the risk of massive O_2_^−^ generation^28^. As the only O_2_^−^ scavenging enzyme, the up-regulation of SOD is indispensable. This allows cyanobacteria to convert O_2_^−^ into H_2_O_2_ and O_2_, while the H_2_O_2_ can be further quenched by various catalases and peroxidases^28^, such as KatG (Fig. 7c). In fact, SOD induction in response to P limitation could be a common strategy amongst photosynthetic organisms since a similar phenomenon is widely observed in cyanobacteria^42^, a marine diatom^43^, and land plants^44^. It should be noted that SOD induction could be extremely important for cyanobacteria that grow under Fe-deficient conditions because O_2_^−^ damage occurs primarily at PSI^45^. PSI content of cyanobacteria decreases significantly under Fe-deficient conditions, and thus up-regulation of SOD may be necessary for maintaining the activity of the limited amount of PSI.

Expression of *sodB* and other Fe-demanding genes is typically repressed by a small regulatory RNA IsaR1^46^ when iron is limiting in *Synechocystis* 6803. Overexpression of *sodB* significantly reduced the intracellular ROS content of Fe-limited cyanobacteria (Supplementary Fig. 11b), but an increased amount of this Fe-dependent metalloenzyme could result in an additional Fe demand, which could account for the decreased growth rate. In addition to SodB, several other Fe-containing proteins were also found to be induced by P deficiency. For example, *ssl2559*, a gene encoding a plant-type ferredoxin, was upregulated under P limitation (Supplementary Fig. 16). *ssl2559* is located downstream of the *sll1291* cluster, and its induction was reduced substantially in the *phoB* mutant, suggesting that this gene may be a part of the same operon. In contrast, although the expression of *katG*, an important Fe-dependent catalase-peroxidase gene, was also significantly upregulated under both +Fe−P (FC = 1.77, *P* < 0.05) and −Fe−P conditions (FC = 1.95, *P* < 0.05), the putative PhoB binding site was absent from its promoter region, suggesting a PhoB-independent regulation. This hypothesis was further supported by qRT-PCR assay showing that all analyzed PhoB targets were significantly upregulated in the Pho regulon-activated PhoR^T412N^ strain^34^, whereas the transcription level of *katG* resembled that of the WT (Supplementary Fig. 17).

P-limitation may increase cellular Fe requirements through multiple pathways. For example, the most widespread alkaline phosphatases among marine microbial communities, PhoD and PhoX, are Fe-dependent^3^. Nutrient amendment experiments performed in the Fe-poor western North Atlantic Ocean showed that the alkaline phosphatase activity of the community responded positively to Fe additions, implicating a role of Fe in P acquisition^47^. The phenomenon of increased Fe content under P deficiency, shown here (Supplementary Fig. 18), is widely reported in photosynthetic organisms^24,44,48^. Thus, regulation of a Fe uptake gene by PhoB is physiologically necessary, as it allows cyanobacteria to meet their increased Fe requirement under P deficiency.

Up-regulation of SOD was also observed in the omics data of co-limited *Trichodesmium*^49^. This, in addition to our metatranscriptomic analysis, indicates a widespread positive response of these genes to P deficiency. Indeed, a survey of the promoter regions of different *sod* isoforms from representative cyanobacteria showed that putative PhoB binding sites are widely present (Supplementary Table 1). A similar observation was also made when analyzing the promoter regions of the orthologues of porin coding genes (Supplementary Table 2). The presence of the putative PhoB binding sites in these species is not only indicative of a direct regulation by PhoB, but also suggests that the PhoB-mediated Fe/P co-limitation acclimation network is conserved within cyanobacteria. Among these species, *Prochlorococcus* and *Synechococcus* are globally important primary producers whose cell sizes are close to the estimated minimum threshold^18^. The presence of a cell-size independent strategy for Fe/P co-limitation acclimation could enable these small-sized cyanobacteria to survive and maintain metabolic activities in oligotrophic oceans.

Interestingly, all the strategies described above likely evolved as adaptations to low-P stress, but are beneficial in dealing with the challenges of Fe-limitation. In fact, Fe and P are deeply interconnected elements. In terrestrial environments, phosphate is naturally associated with ferric oxides, forming insoluble complexes and thus reducing the bioavailability of both nutrients^4^. The highly episodic aerosol dust deposition in marine ecosystems may also lead to a simultaneous deficiency or enrichment of these two elements^9,50,51^. At the cellular level, mineral bio-dissolution by natural cyanobacterial colonies could enable them to co-acquire Fe and P^5^. And the intracellular storage of Fe in cyanobacteria and higher plants is also tightly associated with P^52^. Previous research in *Arabidopsis* reveals that the phosphate-relevant transcription factor, AtPHR1, directly regulates Fe homeostasis through interaction with the promoter regions of the Fe storage gene^53^. Such an interaction was not observed in our Y1H experiment (Supplementary Fig. 19) and ChIP-seq data (Supplementary Data 1), possibly due to subtle differences in the two regulatory systems. However, our study demonstrates that PhoB can specifically bind to the promoter region of a Fe-selective porin (Fig. 6a, b), indicating its critical role in controlling both phosphate and iron homeostasis. Recent research in *Synechocystis* highlighted the crosstalk between the Fe and P stress responses, and an epistatic FtsH1/3-mediated degradation was demonstrated to regulate iron and phosphate regulatory circuits^54^. The existence of crosstalk between the Fe and P stress responses may enable cyanobacteria to optimize resource allocation for maximum growth in nutrient-fluctuating environments.

**Fig. 8:**
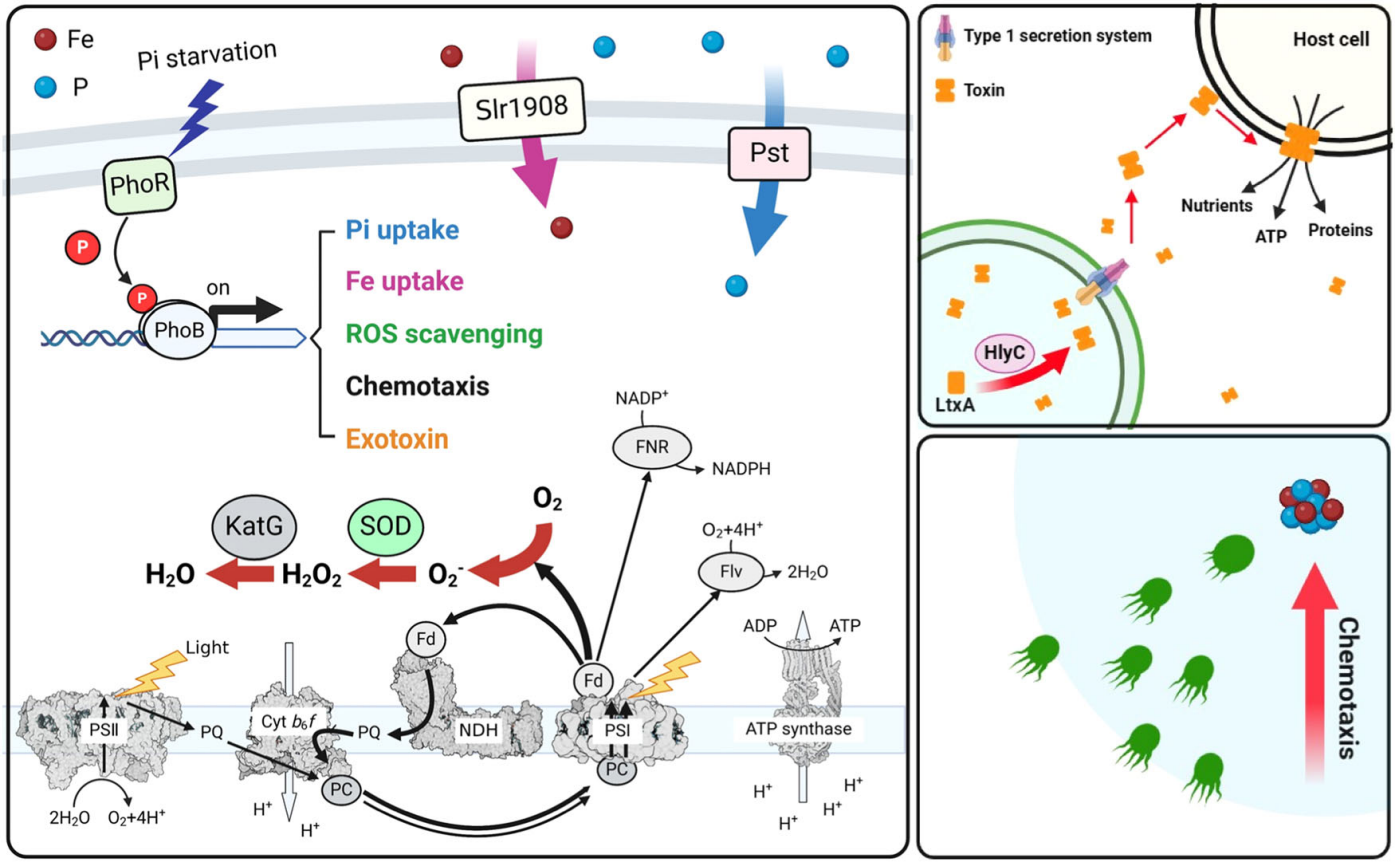
A model of PhoB-mediated Fe/P co-limitation acclimation network. The cellular components potentially regulated by PhoB are shown in color. Transport pathways and reactions up-regulated under Fe/P co-limitation conditions are shown in bold arrows. The figure was created with BioRender.com, and released under a Creative Commons Attribution-NonCommercial-NoDerivs 4.0 International license.

In addition to *slr1908*, a gene encoding the substrate-binding protein of the Fe-siderophore transport system, *sll1202*, was also identified as a putative target of PhoB (Supplementary Data 1). It should be noted that *Synechocystis* 6803 cannot synthesize any siderophores itself; the upregulation of this Fe-siderophore transport gene could enable them to acquire Fe from exogenous siderophores produced by associated bacteria^55^. Moreover, PhoB was also found to regulate the expression of chemotaxis and exotoxin relevant genes. Although little is known about the structure and mechanism of this RTX toxin, mass spectrometry analysis showed that it represents the most abundant protein in the exoproteome of P-limited cyanobacteria (Supplementary Table 3). The strong induction of this virulence factor and enhanced motility via chemotaxis may provide a competitive advantage to Fe-limited cyanobacteria in the natural environments. Based on our findings, a PhoB-centered Fe/P co-limitation acclimation model is proposed (Fig. 8).

It has been reported that the starvation response in *Escherichia coli* is activated when the extracellular Pi concentration is ~4 μM, which is higher than that in the open ocean^56^. According to this threshold, the response of cyanobacteria to low Fe habitats might be a combined effect of Fur and PhoB regulators in the open ocean, and Fe will become the main limiting factor when enhanced Fe uptake capacity cannot fulfill the additional Fe demand of P-limited cyanobacteria. Recent studies have greatly deepened our understanding of P-sensing pathways. In addition to its role in phosphate deficiency response, PhoB is now known to regulate secondary metabolite production, pathogenesis, and quorum sensing^57,58^. The development of mycorrhizal symbiosis in land plants is also mediated in a phosphate-dependent manner^59^. In natural environments, cyanobacteria recruit phycospheric bacteria based on their own needs^55,60^. These associated microbes may help to increase the bioavailability of Fe and P, as several bacteria are known to produce ligands or redox-reactive antibiotics to solubilize mineral dust under P-limitation^61,62^. Whether PhoB also promotes Fe/P co-limitation acclimation by controlling the mutualistic interactions between cyanobacteria and their associated microbes warrants further investigation.

Collectively, our results show that phosphorus deficiency increases the fitness of Fe-limited cyanobacteria through a PhoB regulatory network. Bioinformatic analysis confirms that the identified regulatory pathway is likely to be prevalent among cyanobacteria, suggesting its widespread role in acclimation to Fe/P co-limitation. Given the positive effect of P limitation, the constraint of iron deficiency on oceanic primary productivity might be overestimated. The Fe:P supply ratio might be more important in determining biological production than the concentration of either nutrient alone. This could be extremely important for future simulation and modeling, as P limitation is likely to intensify under global warming in both terrestrial and oceanic environments^63,64^.

## Methods

### Culture conditions and general methods

*Synechocystis* sp. PCC 6803 was grown in YBG11 medium at 30 °C under 30 μmol photons m^−2^  s^−1^. In our study, co-limitation was defined as the growth response of *Synechocystis* to simultaneous low Fe and P concentrations, either of which would be growth-limiting alone. We examined the interactive effects of Fe and P limitation on the growth of the *Synechocystis* in four independent treatments: (1) nutrient replete, 175 µM PO_4_^3−^ and 6 µM Fe; (2) P-limited, 3.5 µM PO_4_^3−^ and 6 µM Fe; (3) Fe-limited, 175 µM PO_4_^3−^ and 0 µM Fe (no added Fe); and (4) Fe/P co-limited, 3.5 µM PO_4_^3−^ and 0 µM Fe. Trace amount of Fe could be left in the Fe-limited medium, and the final concentration of EDTA was 16 µM irrespective of Fe conditions. Before transfer into each treatment, *Synechocystis* cells were pre-adapted in PO_4_^3−^-omitted medium for 4 days to deplete intracellular P.

The growth of each sample was determined by flow cytometry (BD FACSVerse), and data analysis was performed using FlowJo software. Specific growth rates were calculated using the equation *µ* = (ln *N*_*1*_ – ln *N*_*0*_)/*t*, where *N* refers to cell numbers and *t* is time in days^17^. The variation in cell size was determined by estimation from the FSC value of FACS data or by measuring diameters of 150 cells at 400 × magnification with an Olympus BX50 microscope. Absorption spectra of cells were measured using a Specord 210 plus spectrophotometer. Chlorophyll *a* content was determined spectrophotometrically at OD_665_ in methanol extracts and calculated with the formula chlorophyll *a* (mg L^−1^) = 12.6 × OD_665_.

### Mutant construction

The mutant was constructed using standard homologous recombination methods^37^. Taking *phoB* knockout mutant as an example, the coding region of *slr0081* was amplified with specific primers, and an EcoRV restriction site was introduced to the middle of the fragment via fusion PCR. After cloning into the pMD18-T vector, the plasmid was digested with EcoRV and then ligated with a kanamycin resistance cassette. The resulting plasmid was used to transform *Synechocystis* 6803. For ChIP-seq analysis, a 3 × Flag tag was fused to the C-terminal of *phoB*, and the resulting fragment was inserted at the native *phoB* locus. The complete segregation of Δ*phoB* and *phoB*-Flag_3_ was confirmed by PCR (Supplementary Fig. 8).

To construct the overexpression strains, genomic fragments harboring *sodB* and *slr1908* genes were amplified by PCR and introduced into the native locus of *slr0168* and *slr2031*, respectively. The genes were expressed under the control of the modified P*psbAII* promoter^65^. Pho regulon-activated strains were constructed as previously described^34^ with some modifications. Briefly, a gene fragment encoding PhoR^T412N^ was introduced into the P*psbAII* expression vector pHS298^65^. The resulting plasmids were transformed into *Synechocystis* 6803, and complete segregation of the mutant was confirmed by PCR (Supplementary Fig. 17). The primers used are listed in Supplementary Table 4, and the resulting plasmids and strains are listed in Supplementary Table 5.

### Measurement of chlorophyll fluorescence

Chlorophyll fluorescence measurements were performed with a WATER-PAM fluorometer (Walz GmbH, Germany). Before measurements, all samples were kept in the dark for 10 min. The maximal fluorescence (F_m_) was measured using illumination with pulses of red saturating light. The maximal PSII quantum yield (*F*_*v*_/*F*_*m*_) was measured and calculated as (*F*_*m*_ − *F*_*0*_)/*F*_*m*_. Relative ETR was determined according to the standard method^66^. The actinic light intensity was increased stepwise from 0 – 1017 μmol photons m^−2^  s^−1^.

### Total RNA extraction and qRT-PCR analyses

Approximately 100 mL of exponential growth cultures were harvested by centrifugation and frozen in liquid nitrogen. Total RNA was extracted using a TRIzol reagent kit (Invitrogen)^37^. The genomic DNA digestion and cDNA synthesis were performed using the PrimeScript reagent gDNA eraser kit (Takara) according to the manufacturer’s instructions. The transcript abundance of each gene was quantified by qRT-PCR using a 7900HT Fast real-time PCR system (Thermo Fisher, USA), and *rnpB* was used as the reference control. All primers used are listed in Supplementary Table 4.

### Protein preparation and immunoblotting analysis

*Synechocystis* cells grown under different nutrient availabilities were collected and resuspended in ice-cold extract buffer (50 mM Mes-NaOH, 10 mM MgCl_2_, 5 mM CaCl_2_, 25% glycerol, pH 6.5). After cell lysis by ultrasonication, the sample was centrifuged at 3000 *g*, 4 °C, for 10 min to remove the debris and unbroken cells, and the supernatant containing the total protein fraction was collected. The crude membranes containing thylakoid membrane proteins were isolated by further centrifugation at 16 000 *g*, 4 °C, for 1 h. After denaturation at 70 °C for 10 min, the proteins were separated by SDS-PAGE and immunoblotted with corresponding antibodies. The hybridized proteins were visualized with alkaline phosphatase-conjugated secondary antibody (Proteintech) with nitro blue tetrazolium (NBT) and 5-bromo-4-chloro-3-indolylphosphate as the substrates. Uncropped scans of blots and gels are provided in Source Data file.

### Determination of ROS content and SOD activity

To determine the intracellular ROS content, 2 mL of cells were collected and resuspended in 2 mL TE buffer (10 mM Tris-base, 1 mM EDTA-Na_2_, 0.5 mM NaCl, pH 8.0). The samples were then incubated with 10 μM DCFH-DA (Sigma-Aldrich) at 30 °C for 1 h^67^. After the incubation, cells were washed twice with fresh medium and finally resuspended in 1 mL of fresh medium. The fluorescence intensity of the samples was determined with an F-4500 fluorescence spectrophotometer (Hitachi, Japan) using 488 nm excitation and detecting emission at 525 nm.

The SOD activity was detected via in-gel activity staining as described previously^68^. Equal amounts of proteins were loaded and separated by native PAGE at 4 °C. The gel was soaked firstly in phosphate buffer (20 mM NaH_2_PO_4_, 20 mM K_2_HPO_4_, pH 7.8) with 2.5 mM NBT for 30 min and then in phosphate buffer containing 28 mM TEMED and 28 mM flavin for another 20 min. After incubation, the gel was rinsed twice with phosphate buffer and then exposed to white light at 100 µmol photons m^−2^  s^−1^. The result was quantified using ImageJ software.

### Determination of intracellular Fe content

Intracellular Fe quota was measured as described previously^37^. Briefly, 100 ml of *Synechocystis* cells grown under different Fe and P availabilities for 4 days were collected and washed twice with MES-EDTA solution (20 mM MES, 10 mM EDTA, pH 5.0) to remove extracellular Fe. Samples were mixed with HNO_3_ and digested at 180 °C for 25 min. The digested materials were diluted with Milli-Q water (18.2 M Ω cm^−1^). Metal quotas were determined using inductively coupled plasma mass spectrometry (Thermos Scientific), and the result was normalized to dry weight.

### Y1H library screening

Transcription factors that could interact with target promoters were identified by the Y1H system^69^. Taking *sodB* as an example, the predicted promoter region of *sodB* was cloned into the pAbAi vector, and the resulting plasmid was transformed into the Y1HGold yeast bait strain. An efficient Y1H library composed only of transcription factors of *Synechocystis* 6803 was transformed into the Y1H Gold strain obtained above, and the pGADT7 empty vector was used as a negative control. Growth of the yeast cells on the −Leu plates with 200 μg L^−1^ aureobasidin A (AbA) indicates potential *pro*-*sodB* interacting transcriptional factors in the cell. Genes encoding putative binding proteins were identified by DNA sequencing, and the interaction between the identified transcription factor and *sodB* promoter was further verified by point-to-point Y1H.

### EMSA

EMSA was performed according to the method described by Xu et al.^69^. DNA fragments containing the promoter region of PhoB targets were amplified with biotin-labeled and unlabeled primers, respectively. The GST-tagged PhoB recombinant protein was produced and purified with a GST binding resin (Novagen)^69^. The binding reactions between DNA and protein were performed in the EMSA-binding buffer (10 mM Tris-HCl, 50 mM KCl, 1 mM DTT, 0.1% BSA, 5% glycerol, pH 7.5), and the unlabeled DNA and poly (dI-dC) were added to exclude unspecific binding. The reaction mixtures were separated on 6% polyacrylamide gels and then electro-blotted onto nylon membranes (Millipore). After UV cross-linking for 10 min, the nylon membranes were soaked with 5% skimmed milk and then incubated with HRP-conjugated streptavidin for 30 min. Biotin-labeled fragments were detected by chemiluminescence.

### Identification of extracellular proteins

About 200 mL liquid culture (OD_730_ = 0.4) was centrifuged at 4 000 *g*, 4 °C, for 10 min, and the supernatant containing the extracellular proteins was collected. The supernatant was filtered through 0.45 µm pore size filters to remove remaining cell debris, and the proteins in the filtrate were further concentrated by ultrafiltration using centrifugal filters with a molecular weight cut-off of 5 kDa (Millipore). The concentrated extracellular proteins were precipitated with 10% trichloroacetic acid at 4 °C overnight and then dissolved in 50 mM ammonium bicarbonate. The protein digestion and mass spectrometry analysis were performed as described previously^70^. LC-MS/MS spectra were searched against the protein database of *Synechocystis* in UniProt (https://www.uniprot.org/) using the Proteome discoverer 2.1 software. The FDR (false discovery rate) parameter was set to 0.05 for protein identification.

### Sample preparation and transcriptomic analysis

Triplicate cultures of *Synechocystis* grown under different Fe and P availabilities were collected and quickly frozen in liquid nitrogen. Total RNA was extracted using TRIzol Reagent according to the manufacturer’s instructions (Invitrogen). RNA samples were quantified using an ND-2000 spectrophotometer (NanoDrop) and sent to the Majorbio Co. Ltd. for library preparation and 150-bp paired-end sequencing on an Illumina HiSeq×TEN sequencer. Bioinformatics analysis was performed using a cloud platform (www.majorbio.com) based on the data generated by the Illumina platform. RSEM was used to quantify gene and isoform abundances. The TPM method was used to calculate gene expression levels. Differentially expressed genes were identified by using the DESeq2 packages (http://bioconductor.org/packages/release/bioc/html/DESeq2.html). The complete transcriptome results are summarized in Supplementary Data 2, and genes specifically different expressed under −Fe−P were listed in Supplementary Data 3.

### ChIP seq

The *phoB*-FLAG_3_ strains were grown in phosphate omitted medium to an OD_730_ of 0.5 and then cross-linked by 1% formaldehyde treatment for 15 min at room temperature, followed by quenching with 125 mM glycine. Sample preparation for ChIP-Seq was performed as previously described^71^, and libraries were prepared from two biological replicates. The library sequencing was performed with Illumina NovaSeq 6000 by IGENEBOOK Biotechnology Co. Ltd. The MACS2 program (version 2.1.1.20160309) was used for peak calling, and the data were visualized by IGV (version 2.16.0).

### In silico analysis of Pho regulon

The expression and distribution of cyanobacterial *sods* in the surface ocean were determined by analyzing their presence in the global marine TARA metagenome and metatranscriptome datasets^31^. The reference proteins of each SOD isoform were chosen from that of representative species (*sodN* in *Synechococcus* sp. WH8102, *sodC* in *Synechococcus* sp. WH7803, and *sodB* in *Synechocystis* sp. PCC 6803), and the reads recruitment was performed using a cut-off *E*-value < 10^−10^. The source data are provided as a Source Data file.

The conserved PhoB binding motif was extracted from promoter sequences of experimentally verified PhoB target genes using the MEME Suite web server (version 5.5.3; default parameters)^72^. The result was presented as a logo generated by Weblogo (https://weblogo.berkeley.edu/logo.cgi). FIMO web server was utilized to scan for the possible PhoB binding sites with the motif constructed above^72^. The promoter sequences of *sods* and porin genes were extracted from genomes of *Crocosphaera watsonii* WH8501, *Microcystis aeruginosa* NIES-843, *Prochlorococcus marinus* MED4, *Prochlorococcus* sp. MIT9301, *Prochlorococcus* sp. MIT9312, *Prochlorococcus* sp. MIT9313, *Synechococcus* sp. CC9605, *Synechococcus* sp. PCC 7002, *Synechococcus* WH7803, *Synechococcus* WH8102, and *Trichodesmium erythraeum* IMS101.

### Statistical analyses

Unless noted otherwise, all experiments were carried out with at least three independent replicates. The statistical calculations were performed using Origin 2021 (Originlab) software. Tukey’s honest significant difference (HSD) test was used to compare mean values of various treatments, and the difference was considered statistically significant when the test yielded a *P*-value < 0.05. The *t-*test was conducted to assay the statistical significance of the difference between the means of two independent groups. Statistical analyses of meta-omics data were tested by Kruskal-Wallis ANOVA.

### Reporting summary

Further information on research design is available in the Nature Portfolio Reporting Summary linked to this article.

### Supplementary information

Supplementary Information

Peer Review File

Description of Additional Supplementary Files

Supplementary Data 1

Supplementary Data 2

Supplementary Data 3

Reporting Summary

### Source data

Source Data

## Data Availability

Data supporting the findings of this work are available in the Supplementary Data and Source Data files. The transcriptomic data generated in this study have been deposited in NCBI SRA database under the accession code PRJNA1080180. The ChIP-Seq data have been deposited in NCBI GEO database under the accession code GSE260812. The proteomics data have been deposited to the ProteomeXchange Consortium under accession code PXD050522. TARA metagenomes and metatranscriptomes were analyzed using the Ocean Gene Atlas portal [https://tara-oceans.mio.osupytheas.fr/ocean-gene-atlas/], and the data generated in this study were provided in the Source Data file. Source data are provided with this paper.

## References

[1] Moore CM (2013). Processes and patterns of oceanic nutrient limitation. Nat. Geosci..

[2] Browning TJ (2017). Nutrient co-limitation at the boundary of an oceanic gyre. Nature.

[3] Duhamel S (2021). Phosphorus as an integral component of global marine biogeochemistry. Nat. Geosci..

[4] Nussaume L, Desnos T (2022). ‘Je t’aime moi non plus’: a love-hate relationship between iron and phosphate. Mol. Plant.

[5] Shaked Y (2023). Co-acquisition of mineral-bound iron and phosphorus by natural *Trichodesmium* colonies. Limnol. Oceanogr..

[6] Behrenfeld MJ, Kolber ZS (1999). Widespread iron limitation of phytoplankton in the south pacific ocean. Science.

[7] Boyd PW (2007). Mesoscale iron enrichment experiments 1993-2005: Synthesis and future directions. Science.

[8] Wu J, Sunda W, Boyle EA, Karl DM (2000). Phosphate depletion in the western north Atlantic ocean. Science.

[9] Mills MM, Ridame C, Davey M, La Roche J, Geider RJ (2004). Iron and phosphorus co-limit nitrogen fixation in the eastern tropical North Atlantic. Nature.

[10] Held NA (2020). Co-occurrence of Fe and P stress in natural populations of the marine diazotroph *Trichodesmium*. Biogeosciences.

[11] Wen Z (2022). Nutrient regulation of biological nitrogen fixation across the tropical western North Pacific. Sci. Adv..

[12] North RL, Guildford SJ, Smith REH, Havens SM, Twiss MR (2007). Evidence for phosphorus, nitrogen, and iron colimitation of phytoplankton communities in lake Erie. Limnol. Oceanogr..

[13] Robe K, Izquierdo E, Vignols F, Rouached H, Dubos C (2021). The coumarins: secondary metabolites playing a primary role in plant nutrition and health. Trends Plant Sci..

[14] Hou E (2020). Global meta-analysis shows pervasive phosphorus limitation of aboveground plant production in natural terrestrial ecosystems. Nat. Commun..

[15] Garcia NS, Fu F, Sedwick PN, Hutchins DA (2015). Iron deficiency increases growth and nitrogen-fixation rates of phosphorus-deficient marine cyanobacteria. ISME J..

[16] Walworth NG (2016). Mechanisms of increased *Trichodesmium* fitness under iron and phosphorus co-limitation in the present and future ocean. Nat. Commun..

[17] Yang N (2022). Molecular mechanisms underlying iron and phosphorus co-limitation responses in the nitrogen-fixing cyanobacterium *Crocosphaera*. ISME J..

[18] Raven JA (1998). The twelfth tansley lecture. Small is beautiful: the picophytoplankton. Funct. Ecol..

[19] Sunda WG, Hardison DR (2010). Evolutionary tradeoffs among nutrient acquisition, cell size, and grazing defense in marine phytoplankton promote ecosystem stability. Mar. Ecol. Prog. Ser..

[20] Marañón E (2013). Unimodal size scaling of phytoplankton growth and the size dependence of nutrient uptake and use. Ecol. Lett..

[21] Bec B, Collos Y, Vaquer A, Mouillot D, Souchu P (2008). Growth rate peaks at intermediate cell size in marine photosynthetic picoeukaryotes. Limnol. Oceanogr..

[22] Raven JA (1994). Why are there no picoplanktonic O_2_ evolvers with volumes less than 10^−19^ m^3^?. J. Plankton Res..

[23] Flombaum P (2013). Present and future global distributions of the marine cyanobacteria *Prochlorococcus* and *Synechococcus*. Proc. Natl Acad. Sci. USA.

[24] Nam HI (2021). Interdependent iron and phosphorus availability controls photosynthesis through retrograde signaling. Nat. Commun..

[25] Huflejt E (1986). Hydroperoxide metabolism in cyanobacteria. Arch. Biochem. Biophys..

[26] Pérez-Pérez ME, Mata-Cabana A, Sánchez-Riego AM, Lindahl M, Florencio FJ (2009). A comprehensive analysis of the peroxiredoxin reduction system in the cyanobacterium *Synechocystis* sp. strain PCC 6803 reveals that all five peroxiredoxins are thioredoxin dependent. J. Bacteriol..

[27] Riediger M, Hernández-Prieto MA, Song K, Hess WR, Futschik ME (2021). Genome-wide identification and characterization of Fur-binding sites in the cyanobacteria *Synechocystis* sp. PCC 6803 and PCC 6714. DNA Res..

[28] Latifi A, Ruiz M, Zhang CC (2009). Oxidative stress in cyanobacteria. FEMS Microbiol. Rev..

[29] Boden JS, Konhauser KO, Robbins LJ, Sánchez-Baracaldo P (2021). Timing the evolution of antioxidant enzymes in cyanobacteria. Nat. Commun..

[30] Qiu BS, Price NM (2009). Different physiological responses of four marine *Synechococcus* strains (cyanophyceae) to nickel starvation under iron-replete and iron-deplete conditions. J. Phycol..

[31] Vernette C (2022). The Ocean Gene Atlas v2.0: online exploration of the biogeography and phylogeny of plankton genes. Nucleic Acids Res..

[32] Thomas DJ, Avenson TJ, Thomas JB, Herbert SK (1998). A cyanobacterium lacking iron superoxide dismutase is sensitized to oxidative stress induced with methyl viologen but is not sensitized to oxidative stress induced with norflurazon. Plant Physiol..

[33] Suzuki S, Ferjani A, Suzuki I, Murata N (2004). The SphS-SphR two component system is the exclusive sensor for the induction of gene expression in response to phosphate limitation in *Synechocystis*. J. Biol. Chem..

[34] Juntarajumnong W, Hirani TA, Simpson JM, Incharoensakdi A, Eaton-Rye JJ (2007). Phosphate sensing in *Synechocystis* sp. PCC 6803: SphU and the SphS-SphR two-component regulatory system. Arch. Microbiol..

[35] Fitzgerald DM, Stringer AM, Smith C, Lapierre P, Wadea JT (2023). Genome-wide mapping of the *Escherichia coli* PhoB regulon reveals many transcriptionally inert, intragenic binding sites. mBio.

[36] Riediger M (2019). Biocomputational analyses and experimental validation identify the regulon controlled by the redox-responsive transcription factor RpaB. iScience.

[37] Qiu GW (2021). A unique porin meditates iron-selective transport through cyanobacterial outer membranes. Environ. Microbiol..

[38] Su Z, Olman V, Xu Y (2007). Computational prediction of Pho regulons in cyanobacteria. BMC Genom..

[39] Linhartová I (2010). RTX proteins: a highly diverse family secreted by a common mechanism. FEMS Microbiol. Rev..

[40] Balashova NV, Diaz R, Balashov SV, Crosby JA, Kachlany SC (2006). Regulation of *Aggregatibacter* (*Actinobacillus*) *actinomycetemcomitans* leukotoxin secretion by iron. J. Bacteriol..

[41] Carstensen A (2018). The impacts of phosphorus deficiency on the photosynthetic electron transport chain. Plant Physiol..

[42] Tetu SG (2009). Microarray analysis of phosphate regulation in the marine cyanobacterium *Synechococcus* sp. WH8102. ISME J..

[43] Feng TY (2015). Examination of metabolic responses to phosphorus limitation via proteomic analyses in the marine diatom *Phaeodactylum tricornutum*. Sci. Rep..

[44] Misson J (2005). A genome-wide transcriptional analysis using *Arabidopsis thaliana* Affymetrix gene chips determined plant responses to phosphate deprivation. Proc. Natl Acad. Sci. USA.

[45] Herbert SK, Samson G, Fork DC, Laudenbach DE (1992). Characterization of damage to photosystems I and II in a cyanobacterium lacking detectable iron superoxide dismutase activity. Proc. Natl Acad. Sci. USA.

[46] Georg J (2017). Acclimation of oxygenic photosynthesis to iron starvation is controlled by the sRNA IsaR1. Curr. Biol..

[47] Browning TJ (2017). Iron limitation of microbial phosphorus acquisition in the tropical North Atlantic. Nat. Commun..

[48] Zheng L (2009). Physiological and transcriptome analysis of iron and phosphorus interaction in rice seedlings. Plant Physiol..

[49] Cerdan-Garcia E (2022). Transcriptional responses of *Trichodesmium* to natural inverse gradients of Fe and P availability. ISME J..

[50] Letelier RM (2019). Climate-driven oscillation of phosphorus and iron limitation in the North Pacific Subtropical Gyre. Proc. Natl Acad. Sci. USA.

[51] Wang S (2022). Colonies of the marine cyanobacterium *Trichodesmium* optimize dust utilization by selective collection and retention of nutrient-rich particles. iScience.

[52] Laulhere JP, Laboure AM, Van Wuytswinkel O, Gagnon J, Briat JF (1992). Purification, characterization and function of bacterioferritin from the cyanobacterium *Synechocystis* PCC 6803. Biochem. J..

[53] Bournier M (2013). *Arabidopsis* ferritin 1 (AtFer1) gene regulation by the phosphate starvation response 1 (AtPHR1) transcription factor reveals a direct molecular link between iron and phosphate homeostasis. J. Biol. Chem..

[54] Krynická V (2019). Depletion of the FtsH1/3 proteolytic complex suppresses the nutrient stress response in the cyanobacterium *Synechocystis* sp strain PCC 6803. Plant Cell..

[55] Qiu GW, Koedooder C, Qiu BS, Shaked Y, Keren N (2022). Iron transport in cyanobacteria—from molecules to communities. Trends Microbiol..

[56] Karl DM (2014). Microbially mediated transformations of phosphorus in the sea: New views of an old cycle. Annu. Rev. Mar. Sci..

[57] Santos-Beneit F (2015). The Pho regulon: a huge regulatory network in bacteria. Front. Microbiol..

[58] Sola-Landa A, Moura RS, Martin JF (2003). The two-component PhoR-PhoP system controls both primary metabolism and secondary metabolite biosynthesis in *Streptomyces lividans*. Proc. Natl Acad. Sci. USA.

[59] Shi J (2021). A phosphate starvation response-centered network regulates mycorrhizal symbiosis. Cell.

[60] Zhao L (2023). The facilitating role of phycospheric heterotrophic bacteria in cyanobacterial phosphonate availability and *Microcystis* bloom maintenance. Microbiome.

[61] Romano S, Bondarev V, Kölling M, Dittmar T, Schulz-Vogt HN (2017). Phosphate limitation triggers the dissolution of precipitated iron by the marine bacterium *Pseudovibrio* sp. FO-BEG1. Front. Microbiol..

[62] Mcrose DL, Newman DK (2021). Redox-active antibiotics enhance phosphorus bioavailability. Science.

[63] Tian Y (2023). Long-term soil warming decreases microbial phosphorus utilization by increasing abiotic phosphorus sorption and phosphorus losses. Nat. Commun..

[64] Doney SC (2006). Plankton in a warmer world. Nature.

[65] Jiang HB, Lou WJ, Du HY, Price NM, Qiu BS (2012). Sll1263, a unique cation diffusion facilitator protein that promotes iron uptake in the cyanobacterium *Synechocystis* sp. strain PCC 6803. Plant Cell Physiol..

[66] Xu M (2008). Properties of mutants of *Synechocystis* sp. strain PCC 6803 lacking inorganic carbon sequestration systems. Plant Cell Physiol..

[67] Qiu GW, Lis H, Qiu BS, Keren N (2021). Long-term iron deprivation and subsequent recovery uncover heterogeneity in the response of cyanobacterial populations. Environ. Microbiol..

[68] Ke WT, Dai GZ, Jiang HB, Zhang R, Qiu BS (2014). Essential roles of iron superoxide dismutase in photoautotrophic growth of *Synechocystis* sp. PCC 6803 and heterogeneous expression of marine *Synechococcus* sp. CC9311 copper/zinc superoxide dismutase within its *sodB* knockdown mutant. Microbiology.

[69] Xu HF (2022). Coevolution of tandemly repeated hlips and RpaB-like transcriptional factor confers desiccation tolerance to subaerial *Nostoc* species. Proc. Natl Acad. Sci. USA.

[70] Xu C (2022). Global landscape of native protein complexes in *Synechocystis* sp. PCC 6803. Genom. Proteom. Bioinforma..

[71] Stringer AM (2014). Genome-scale analyses of *Escherichia coli* and *Salmonella enterica* AraC reveal noncanonical targets and an expanded core regulon. J. Bacteriol..

[72] Bailey TL (2009). MEME Ssite: tools for motif discovery and searching. Nucleic Acids Res..

