# Supplementary material for: Phosphorus deficiency alleviates iron limitation in Synechocystis cyanobacteria through direct PhoB-mediated gene regulation
Source: Nat Commun. 2024 May 24;15:4426. doi: 10.1038/s41467-024-48847-4 (PMC11126600; doi:10.1038/s41467-024-48847-4)
Supplement: Supplementary file 1 — Supplementary Information [file 41467_2024_48847_MOESM1_ESM.pdf]

Supplementary Information for

Phosphorus deficiency alleviates iron limitation in *Synechocystis* cyanobacteria through direct PhoB-mediated gene regulation

Guo-Wei Qiu, Wen-Can Zheng, Hao-Ming Yang, Yu-Ying Wang, Xing Qi, Da Huang, Guo-Zheng Dai, Huazhong Shi, Neil M. Price & Bao-Sheng Qiu

Corresponding author: Bao-Sheng Qiu (email: bsqiu@mail.ccnu.edu.cn).

This file includes

Supplementary Figures 1 to **19** and **Supplementary Tables 1** to **5**.

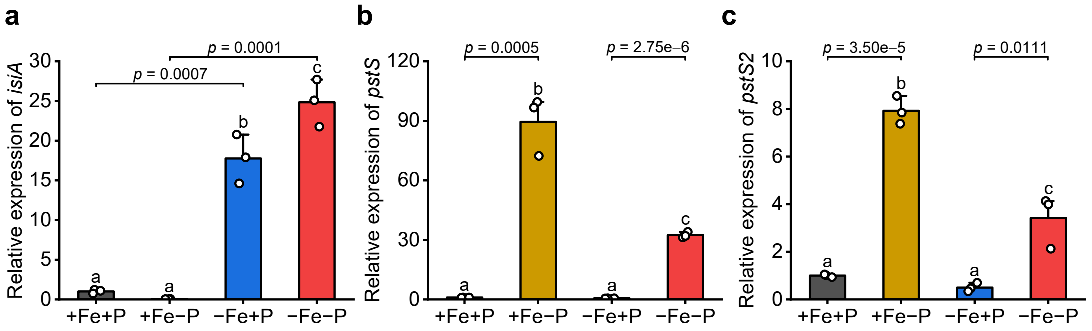

Supplementary Fig. 1 qRT-PCR analysis confirming the −Fe−P samples were co-limited with iron and phosphorus. The *isiA* (**a**) and *pstS* genes (**b-c**) were chosen as biomarkers to indicate Fe and P limitation. Data shown are mean ± SD of three independent biological replicates, and the results were normalized to the transcript levels measured under +Fe+P conditions. Letters above bars indicate statistical significance ($P < 0.05$) calculated by one-way ANOVA across all treatments. Line segments and corresponding *P*-values represent statistical significance calculated by two-sided Student's *t* test. The primers used here are listed in Supplementary Table 4. Source data are provided as a Source Data file.

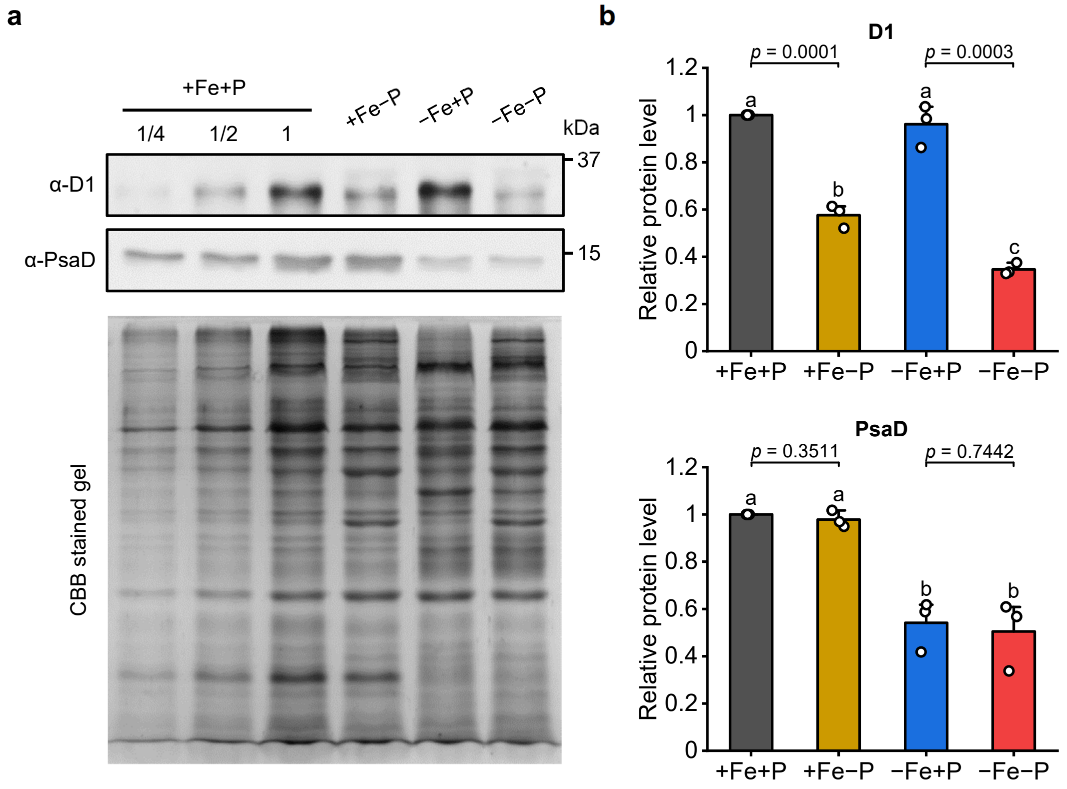

Supplementary Fig. 2 The variation of PSI and PSII content of *Synechocystis* grown under different iron and phosphorus availabilities. a Analysis of thylakoid membrane protein accumulation in *Synechocystis* after four days of growth under +Fe+P, +Fe−P, −Fe+P, and −Fe−P conditions. Membrane proteins of +Fe+P (with series of dilution for protein loading, 10 µg [one-quarter dilution], 20 µg [half dilution], and 40 µg [1, without dilution], respectively), +Fe−P (40 µg), −Fe+P (40 µg), and −Fe−P (40 µg) samples were separated by SDS–PAGE and probed with specific antisera. Similar results were obtained from three independent biological replicates. **b** Proteins immunodetected from (**a**) were quantified with ImageJ software. Data shown are mean ± SD of three independent biological replicates and values are given relative to protein levels of +Fe+P samples. Letters above bars indicate statistical significance ($P < 0.05$) calculated by one-way ANOVA across all treatments. Line segments and corresponding P-values represent statistical significance calculated by two-sided Student's t test. Source data are provided as a Source Data file.

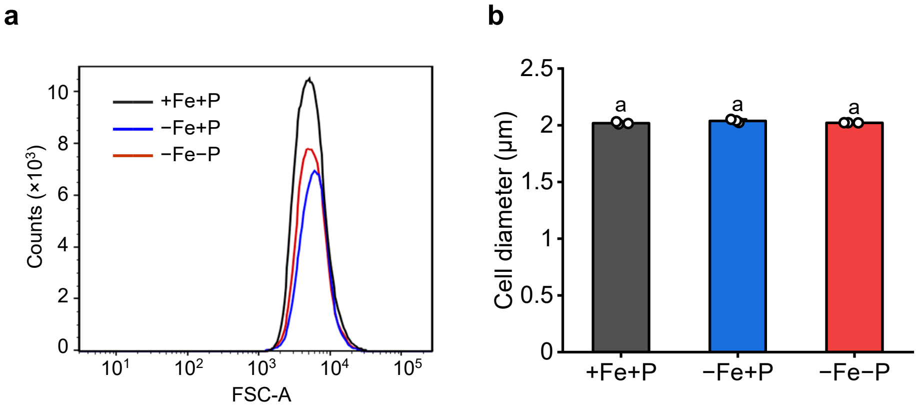

Supplementary Fig. 3 Cell size determined by flow cytometry. a The cell size of *Synechocystis* was indicated as the intensity of forward scatter (FSC). Data shown are from representative sample of three independent biological replicates. **b** The diameter of cells was estimated from linear extrapolation: cell size (μm)=3 + $(F_c - F_3)/(F_3 - F_2)$, where F_c, F_2, and F_3 represent the FSC peak intensities of the cells, 2 μm bead, and 3 μm bead, respectively. Data shown are mean ± SD of three independent biological replicates. Letters above bars indicate statistical significance ($P < 0.05$) calculated by one-way ANOVA across all treatments. Source data are provided as a Source Data file.

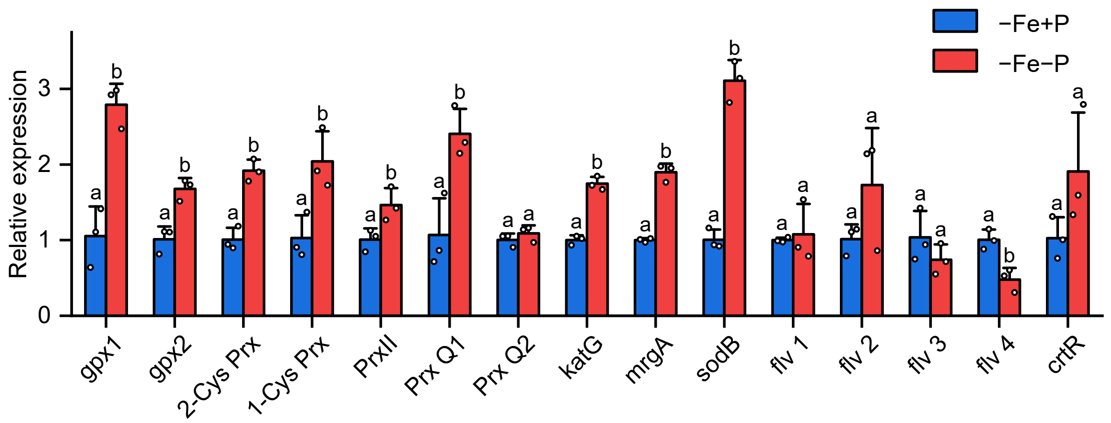

Supplementary Fig. 4 The transcript levels of the genes controlling ROS homeostasis in *Synechocystis*. The expression patterns of the genes encoding glutaredoxins (Gpx1 and Gpx2), thioredoxin (1-Cys Prx, 2-Cys Prx, PrxII, Prx Q1, and Prx Q2), catalase-peroxidase (KatG), superoxide dismutase (SodB), DNA binding protein (MrgA), flavodiiron proteins (Flv1-4), and carotenoid hydroxylase (CrtR) were analyzed by qRT-PCR. Data shown are mean ± SD of three independent biological replicates, and the results were normalized to the transcript levels measured under −Fe+P conditions. The different letters above the bars represent statistical significance ($P < 0.05$, two-sided Student's t test). The primers used here are listed in Supplementary Table 4. Source data are provided as a Source Data file.

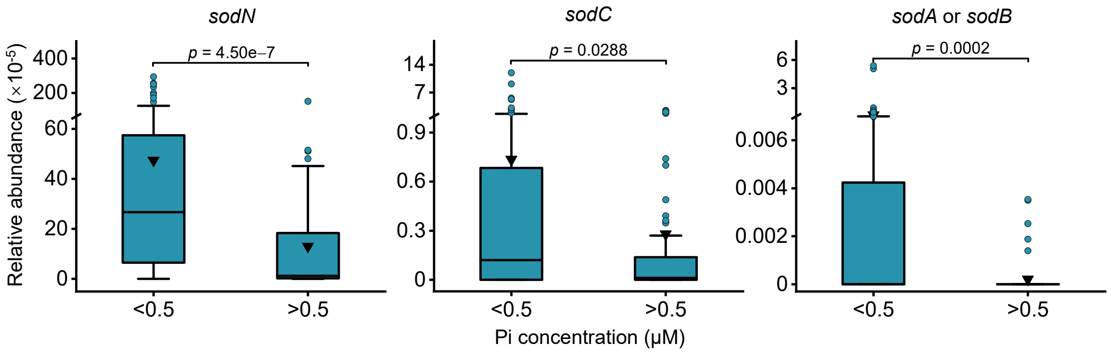

Supplementary Fig. 5 Metatranscriptomic abundance of *sods* under different Pi concentrations. Whisker box plots show the transcript abundance of different *sods* isoforms from global marine TARA datasets. Data are presented by Pi concentrations. The abundance of each *sod* was calculated by dividing the sum of the abundances of their homologs by the sum of total gene abundance from all reads in the sample. The whiskers extend to 1.5× the interquartile range beyond the first and third quartiles (boxes). Exclusive median (line), average (triangle), and atypical values (circles) are also indicated. Line segments and corresponding *P*-values represent statistical significance calculated by Mann-Whitney *U* test. Source data are provided as a Source Data file.

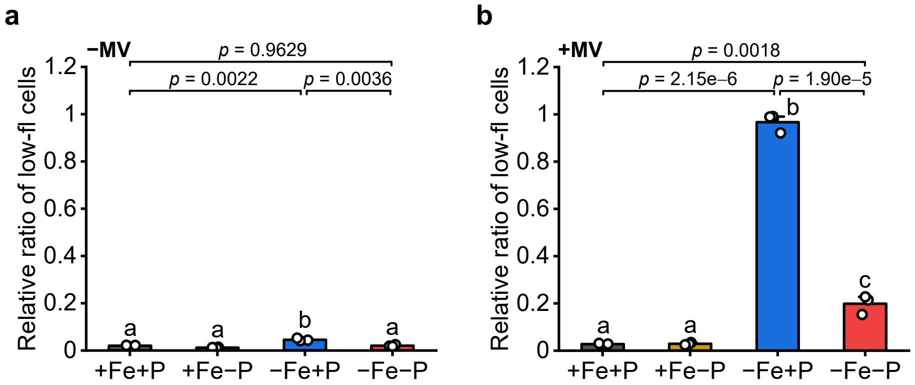

Supplementary Fig. 6 Quantification of the ratio of low-fl cells before and after MV treatment. The change of auto-fluorescence intensity of *Synechocystis* cells grown in the absence (**a**) and presence (**b**) of 0.3 μM MV was analyzed by flow cytometry, and the gating was performed as described in Fig. 4b. Data shown are mean ± SD of three independent biological replicates. Letters above bars indicate statistical significance ($P < 0.05$) calculated by one-way ANOVA across all treatments. Line segments and corresponding P-values represent statistical significance calculated by two-sided Student's t test. Source data are provided as a Source Data file.

a

ID	Pho Boxes	Position
slr1516	ATTAACAAgggATTAAAAAactGTCAGGCT	−174
slr1908	GTTAAGTAaagGTTAAGGAtccGTTAAGCA	−337
sll1291	GATCAACTataGTTAACTTaagATTAAGAA	−87
sll1552	CTTAATTTtaaTTTAACCTtaaTTTAATCT	−698

b

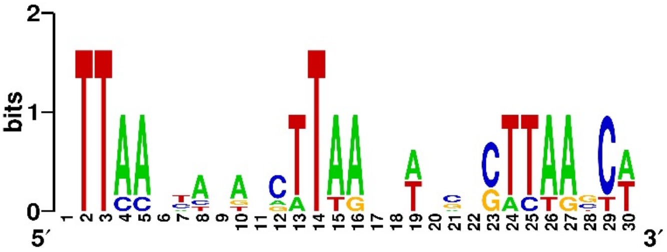

c

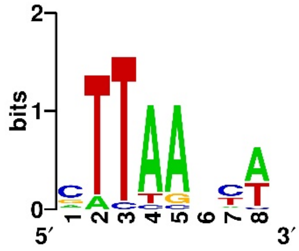

d

	Pho box1		Pho box2		Pho box3
WT:	G**TTAA**GTA	aag	G**TTAA**GGA	tcc	G**TTAA**GCA
M1:	GGGGGGTA	aag	GTTAAGGA	tcc	GTTAAGCA
M2:	GTTAAGTA	aag	GGGGGGGA	tcc	GTTAAGCA
M3:	GTTAAGTA	aag	GTTAAGGA	tcc	GGGGGGCA
Mu:	GGGGGGTA	aag	GGGGGGGA	tcc	GGGGGGCA

PhoB (μM)	0	1.6	3.2	3.2	3.2	3.2	3.2	3.2	3.2	GST
Competitor	−	−	−	−	−	−	−	SC	NSC	−
WT probe	WT	WT	WT	−	−	−	−	WT	WT	WT
Mutant probe	−	−	−	M1	M2	M3	Mu	−	−	−

Shift probe

Free probe

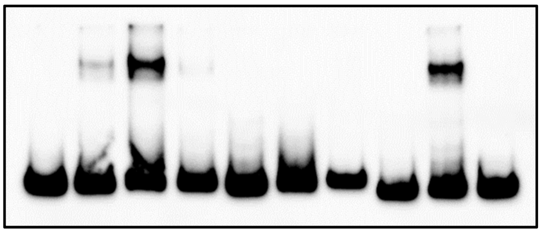

Supplementary Fig. 7 Identification of PhoB binding sequence. a A conserved motif containing three 8 bp tandem repeats with two 3 bp linkers was found in the promoter regions of the newly identified PhoB regulons. Each of the repeats fits well with the consensus sequences of Pho boxes (CTTAACCT). **b** An upgraded PhoB binding motif generated by the Weblogo server based on the promoter sequences of the experimentally verified PhoB targets. **c** Logo representation of the profile of the combined putative Pho boxes of the three tandem repeats shown in panel (**b**). **d** Binding to the TTAA motif was verified by a standard EMSA. Mutated *slr1908* promoter are shown on the top. Similar results were obtained from two independent experiments.

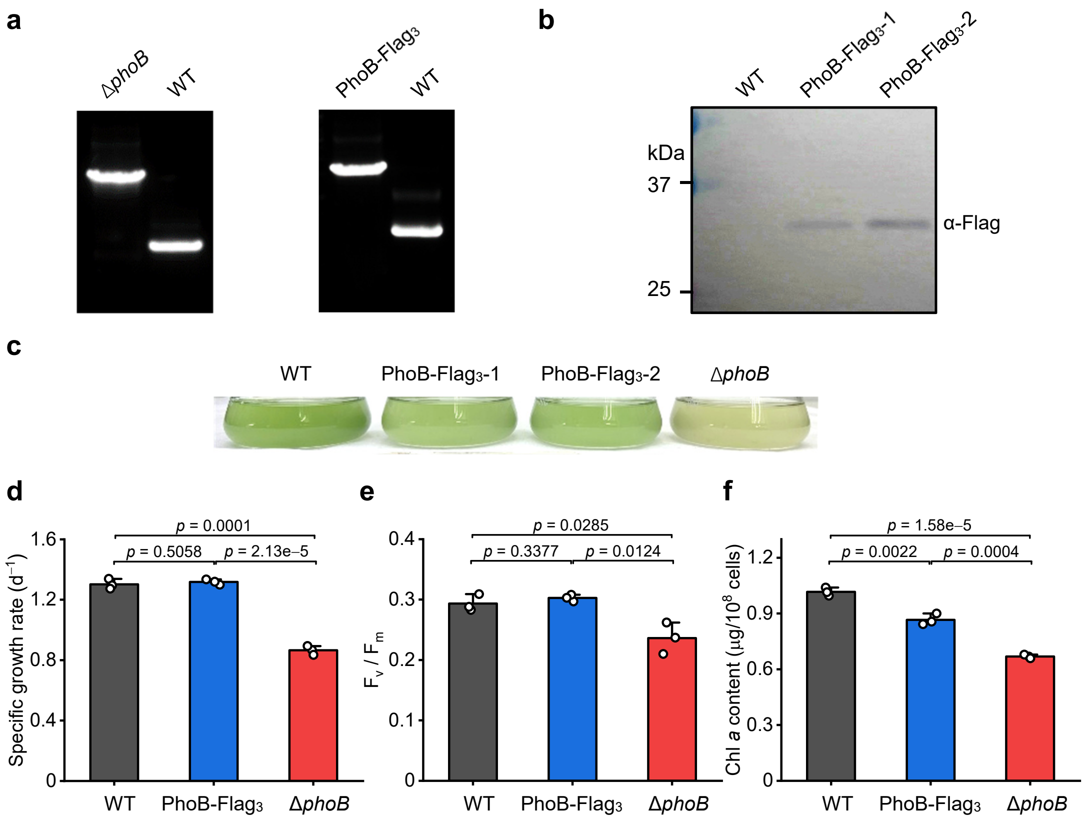

Supplementary Fig. 8 Characterization of *ΔphoB* and *phoB*-Flag3 strains. a PCR results demonstrating that the two strains were completely segregated. The primers used here are listed in Supplementary Table 4. **b** Western blotting analysis showing that the PhoB-Flag3 protein could be specifically recognized by the anti-Flag monoclonal antibodies (Sigma). **c** Image of different *Synechocystis* strains after four days of growth under P-limited conditions. The experiment was repeated with similar results three times. The two *phoB*-Flag3 cultures present here are biological replicates that were used for ChIP-seq analysis. **d**-**f** Growth rates (**d**), Fv/Fm values (**e**), and chlorophyll *a* contents (**f**) of the WT, phoB-Flag3, and Δ*phoB* strains after four days of growth under P-limitation. Data shown are measured from three independent biological replicates. Letters above bars indicate statistical significance ($P < 0.05$) calculated by one-way ANOVA across all treatments. Line segments and corresponding *P*-values represent statistical significance calculated by two-sided Student's *t* test. Source data are provided as a Source Data file.

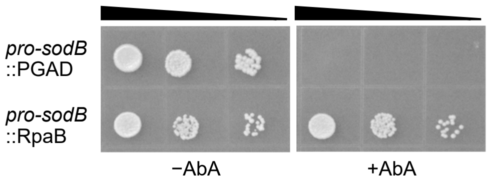

Supplementary Fig. 9 Verification of the interaction between RpaB and the promoter of *sodB*. The interaction between the *cis*-acting element *pro-sodB* and RpaB was demonstrated by Y1H assay.

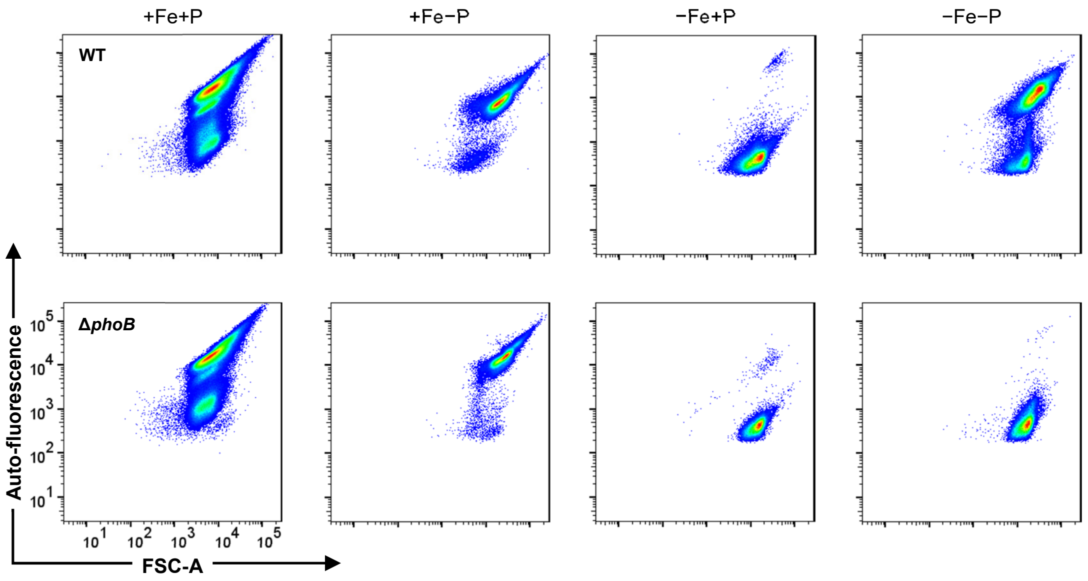

Supplementary Fig. 10 Flow cytometry scatterplots of *Synechocystis* cells under MV treatment. 0.3 μM MV was added into the growth medium to induce O_2^- generation, and the change of auto-fluorescence intensity of *Synechocystis* cells was analyzed by flow cytometry. Data shown are from representative sample of three independent biological replicates. FSC, forward scatter.

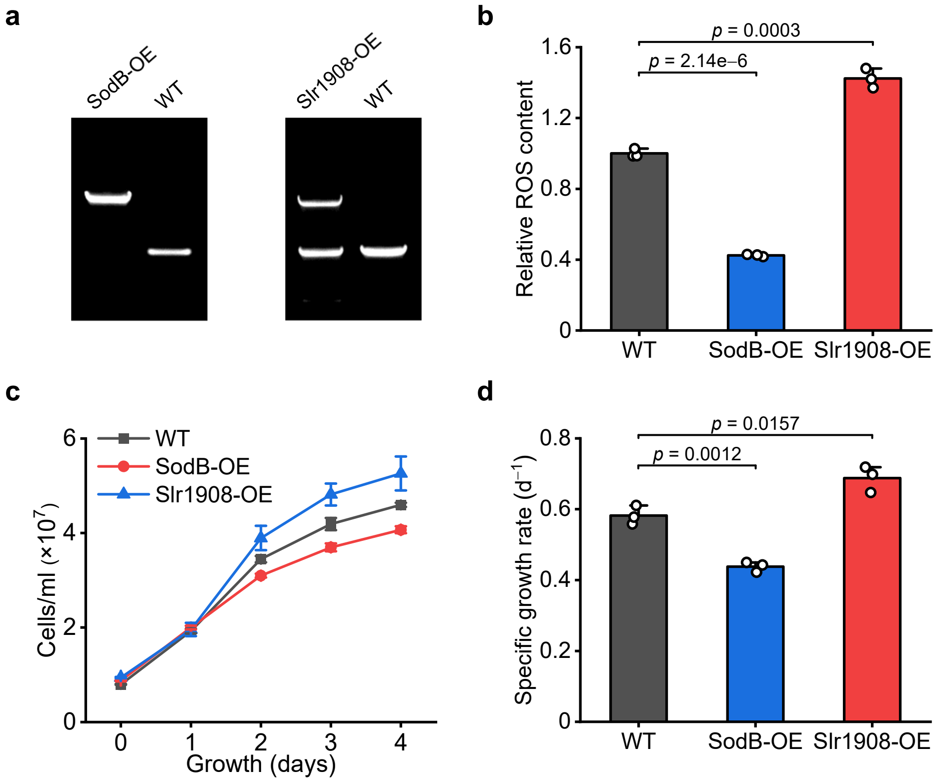

Supplementary Fig. 11 Physiological characterization of SodB-OE and Slr1908-OE strains in *Synechocystis* 6803. a The segregation of SodB-OE and Slr1908-OE strains was analyzed by PCR. The primers used here are listed in Supplementary Table 4. **b** The intracellular ROS accumulation of *Synechocystis* strains after four days of growth under Fe limitation. Values are mean ± SD of three independent biological replicates, and the results were normalized to the ROS level of WT. **c** The growth curve of WT, SodB-OE, and Slr1908-OE strains under Fe-limited conditions. Values are mean ± SD of three independent biological replicates. **d** Growth rates of WT, SodB-OE, and Slr1908-OE strains grown under Fe-limited conditions. Values are mean ± SD of three independent biological replicates. Line segments and corresponding *P*-values represent statistical significance calculated by two-sided Student's *t* test. Source data are provided as a Source Data file.

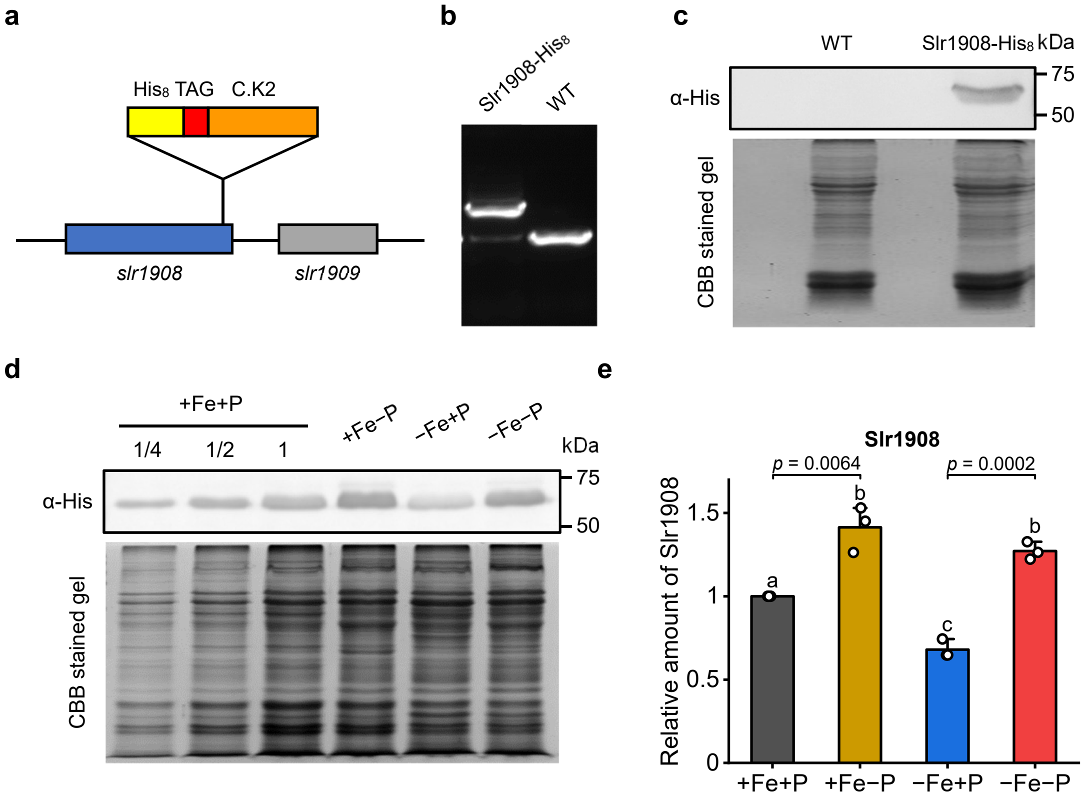

Supplementary Fig. 12 Quantification of the protein content of Slr1908 under different Fe and P availabilities. **a** To probe the expression of Slr1908 at protein level, a C-terminal 8×His tag was introduced at the native *slr1908* locus, and the kanamycin resistance fragment (C.K2) was used as a selectable marker. **b** The segregation of Slr1908-His_8 strains was analyzed by PCR. The primers used here are listed in Supplementary Table 4. **c** Western blotting analysis showing that the Slr1908-His_8 protein could be specifically recognized by the anti-His monoclonal antibodies (Proteintech). **d** Analysis of Slr1908 accumulation in Slr1908-His_8 strains after four days of growth under +Fe+P, +Fe−P, −Fe+P, and −Fe−P conditions. The total proteins of +Fe+P (with series of dilution for protein loading, 5 μg [one-quarter dilution], 10 μg [half dilution], and 20 μg [1, without dilution], respectively), +Fe−P (20 μg), −Fe+P (20 μg), and −Fe−P (20 μg) samples were separated by SDS–PAGE and probed with anti-His monoclonal antibodies. Similar results were obtained from three independent biological replicates. **e** Proteins immunodetected from (**d**) were quantified with ImageJ software. Data shown are mean ± SD of three independent biological replicates and values are given relative to protein levels of +Fe+P samples. Letters above bars indicate statistical significance ($P < 0.05$) calculated by one-way ANOVA across all treatments. Line segments and corresponding P-values represent statistical significance calculated by two-sided Student's t test. Source data are provided as a Source Data file.

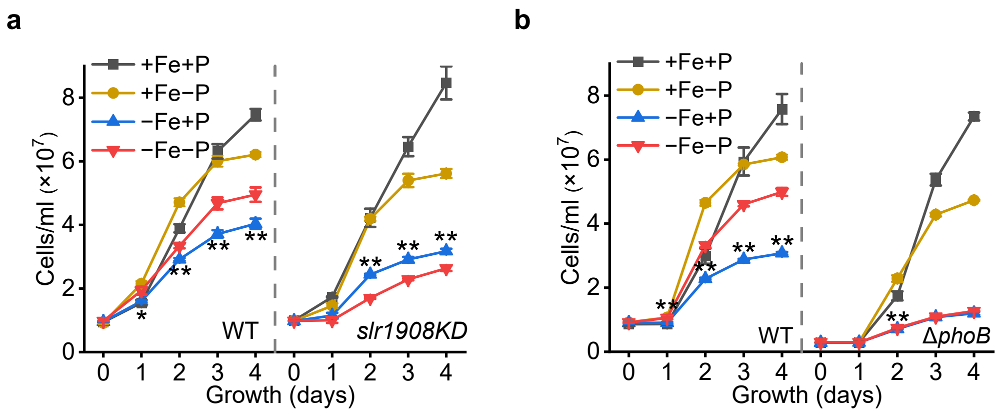

Supplementary Fig. 13 The growth curve of WT, *slr1908KD* (a), and Δ*phoB* (b) under different iron and phosphorus availabilities. Values are mean ± SD of three independent biological replicates. Asterisks indicate a significant difference between −Fe+P and −Fe−P using the two-sided Student's *t* test. **P* < 0.05; ***P* < 0.01. Source data are provided as a Source Data file.

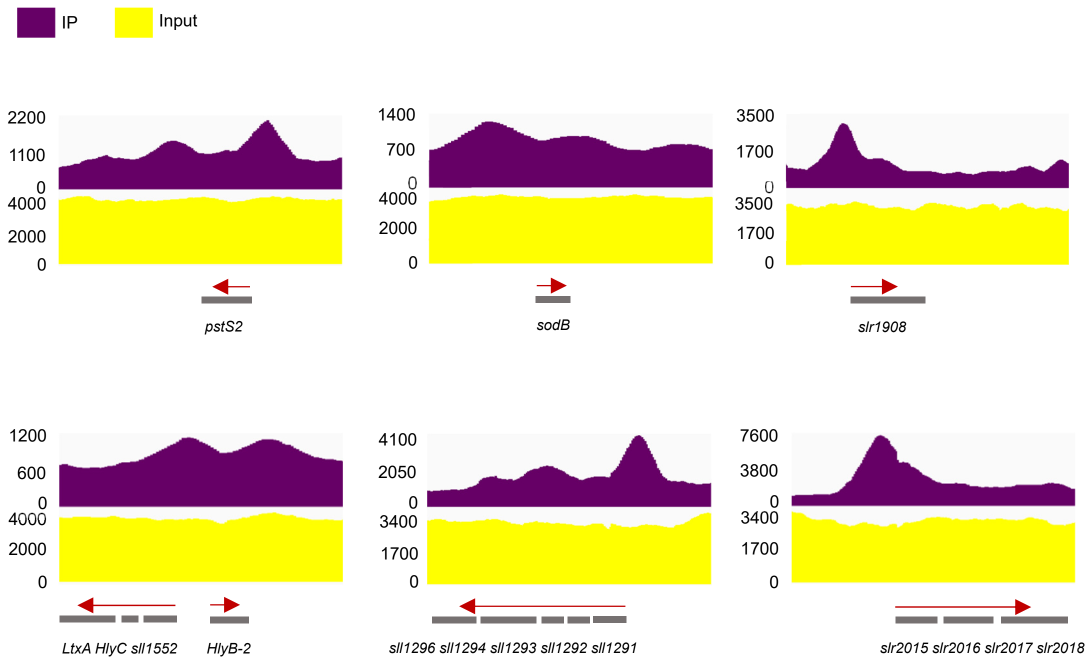

Supplementary Fig. 14 PhoB-binding sites identified by ChIP-Seq. The downstream genes are shown at the bottom and arrows indicate the directions of transcription. The numbers on the Y-axis represent the reads. The peaks were identified using MACS2 program with $q < 0.05$.

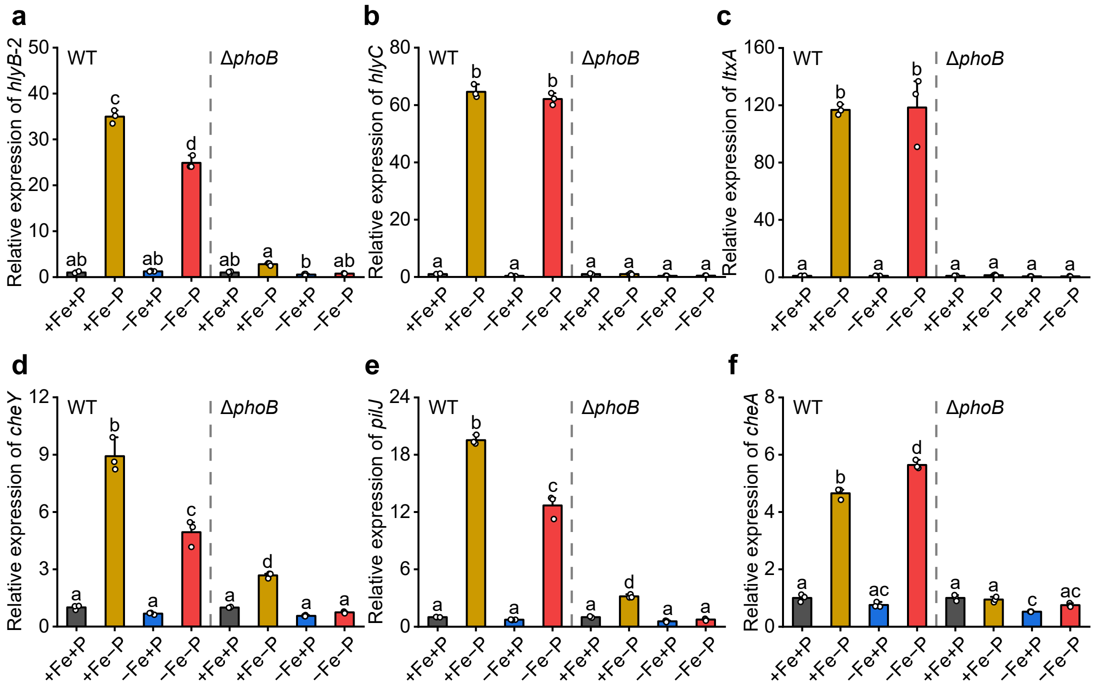

Supplementary Fig. 15 The transcript levels of putative PhoB targets in WT and *phoB* mutants under different Fe and P availabilities. The expression of the genes involved in exotoxin secretion (**a-c**) and chemotaxis (**d-f**) were analyzed by qRT-PCR. Data shown are mean ± SD of three independent biological replicates, and the results were normalized to the transcript levels measured under +Fe+P conditions. Letters above bars indicate statistical significance ($P < 0.05$) calculated by one-way ANOVA across all treatments. The primers used here are listed in Supplementary Table 4. Source data are provided as a Source Data file.

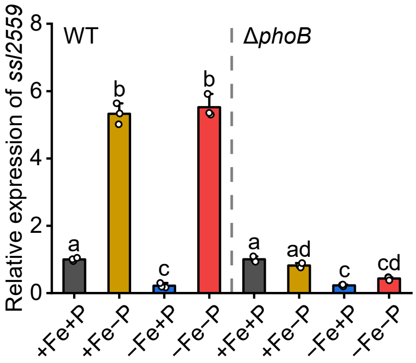

Supplementary Fig. 16 The transcript levels of *ssl2559* in WT and *phoB* mutants under different Fe and P availabilities. Data shown are mean ± SD of three independent biological replicates, and the results were normalized to the transcript levels measured under +Fe+P conditions. Letters above bars indicate statistical significance ($P < 0.05$) calculated by one-way ANOVA across all treatments. The primers used here are listed in Supplementary Table 4. Source data are provided as a Source Data file.

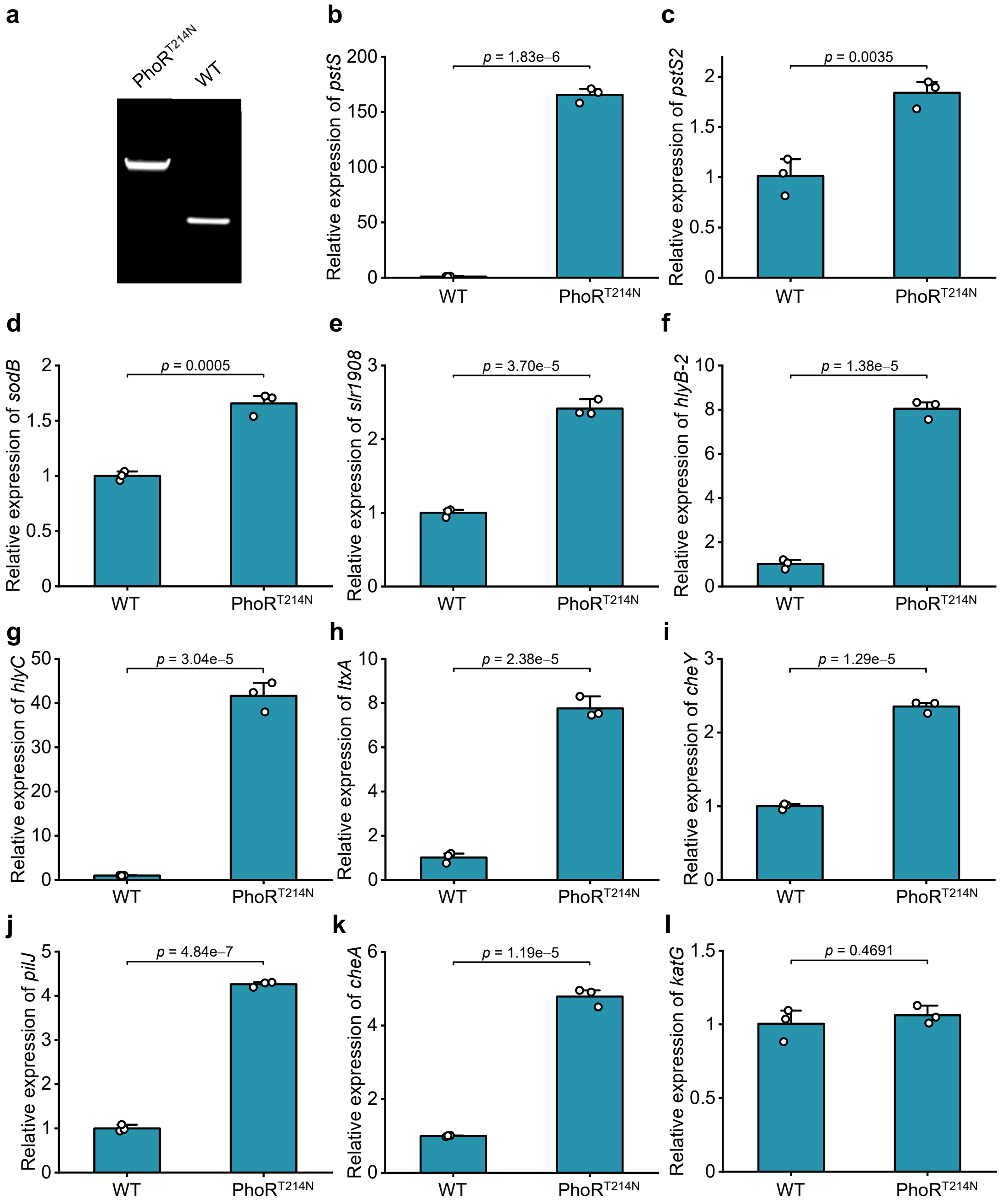

Supplementary Fig. 17 The transcript levels of putative PhoB targets in WT and PhoR^{T214N} strains. a PCR results demonstrating that the PhoR^{T214N} strains were completely segregated. **b-l** Relative mRNA abundance of putative PhoB targets (**b-k**) and *katG* (**l**) in *Synechocystis* strains after four days of growth under Fe-replete conditions. Data shown are mean ± SD of three independent biological replicates, and the results were normalized to the transcript levels measured in the WT. Line segments and corresponding *P*-values represent statistical significance calculated by two-sided Student's *t* test. The primers used here are listed in Supplementary Table 4. Source data are provided as a Source Data file.

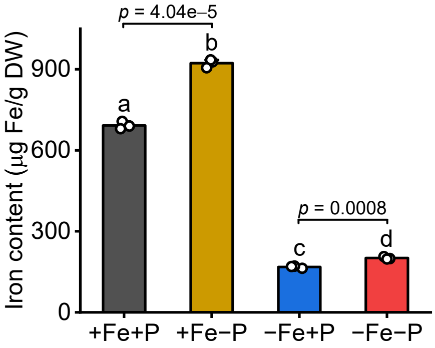

Supplementary Fig. 18 The intracellular Fe content of *Synechocystis* after four days of growth under different iron and phosphorus availabilities. Values are mean ± SD of three independent biological replicates. Letters above bars indicate statistical significance ($P < 0.05$) calculated by one-way ANOVA across all treatments. Line segments and corresponding P-values represent statistical significance calculated by two-sided Student's t test. Source data are provided as a Source Data file.

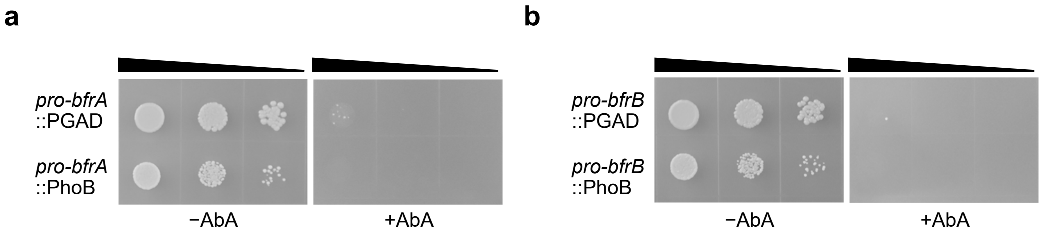

Supplementary Fig. 19 Determining the interactions between PhoB and the promoters of Fe storage genes. The interaction between PhoB and the *cis*-acting elements *pro-bfrA* (**a**) and *pro-bfrB* (**b**) was evaluated by Y1H. No direct interaction was observed under our experiment conditions.

Supplementary Table 1. Predicted Pho boxes present in the promoters of *sod* isoforms.

Gene ID	Isoforms	Matched Sequence	*P*-value	Position	Strains
PMM1294	*sodN*	GTTAATCTacaAGTAAGATataTTCAAATA	8.95E-05	−11	*Prochlorococcus marinus* MED4
CwatDRAFT_2983	*sodN*	GTTAAAGAaaaGTCAGCAAaaaTTTAAAAT	8.95E-05	−141	*Crocosphaera watsonii* WH8501
Tery_4533	*sodA*	ATTAATTCggATAAATTTttaGAGAATTT	8.05E-04	−32	*Trichodesmium erythraeum* IMS101
Tery_0891	*sodN*	TTTAACAAcaaTGTAATTTtttCTATATAA	8.33E-04	−0	*Trichodesmium erythraeum* IMS101
PMT9312_1390	*sodN*	ATTAAAAGtcaATTAAATTaagTAAGAAAA	8.33E-04	−64	*Prochlorococcus* sp. MIT9312
P9301_14791	*sodN*	ATTAAAAGtcaATTAAATTaagTAAGAAAA	8.33E-04	−64	*Prochlorococcus* sp. MIT9301
MAE_53990	*sodB*	TTTAAGGTagtGTTGTTAAcctTTTGTCAG	3.54E-03	−30	*Microcystis aeruginosa* NIES-843
PMT_0340	*sodN*	CTGAAAAGcaaATCAAATCaaaAGCAAACC	4.79E-03	−51	*Prochlorococcus* sp. MIT9313
MAE_16920	*sodA*	TTTATTTTcaaTTAATTATtcgCATAACAA	5.08E-03	−31	*Microcystis aeruginosa* NIES-843
CwatDRAFT_6299	*sodA*	ATTAATAAtggTAATTATTtttCTTAATTT	5.25E-03	−169	*Crocosphaera watsonii* WH8501
SYNW1626	*sodN*	CTTAAAGCgagCCTAGCTGcggCCCATCAC	5.36E-03	−18	*Synechococcus* sp.WH8102
SynWH7803_0951	*sodC*	TTTCATTGtcgTTCAAACGgcgCTAGCGCG	6.28E-03	−17	*Synechococcus* WH7803
SYNPCC7002_A0242	*sodB*	TTTTCGATggaGATAACCGgacTCGAACCG	6.28E-03	−268	*Synechococcus* sp. PCC7002
Syncc9605_0873	*sodN*	GTTCAAAGcggAATTGTCTtggCTTGAGCC	6.28E-03	−2	*Synechococcus* sp. CC9605

Supplementary Table 2. Predicted Pho boxes present in the promoters of porin coding genes.

Gene ID	Matched Sequence	*P*-value	Position	Strains
P9301_12401	ATTAATTAgttCTTAACCTcacCTTAAACT	1.12E-07	−236	*Prochlorococcus* sp. MIT9301
PMT9312_0721	GTTAATTAgttCTTAACCTtctCTTAAAAC	1.12E-07	−236	*Prochlorococcus* sp. MIT9312
PMM0709	ATTAATAGctaATTAATCTtctCTTAAACT	1.64E-07	−241	*Prochlorococcus marinus* MED4
SYNPCC7002_A0782	TTTAACCCcgaCTTCAACTttgGTTAACCT	9.37E-07	−42	*Synechococcus* sp. PCC7002
SYNPCC7002_A1034	TTTAACCTgccATTAATCCaacATCAAGGC	9.37E-07	−211	*Synechococcus* sp. PCC7002
PMT_0998	CTTAAAAAcaaCTTATTGCtctCTTAACCT	2.66E-06	−196	*Prochlorococcus* sp. MIT9313
CwatDRAFT_5912	CTTAACTAaccATTAGATTagtATTAATAG	5.38E-06	−60	*Crocosphaera watsonii* WH8501
P9301_12261	ATTAATCAttaTTTAAATTatcTTAAAATT	6.00E-06	−23	*Prochlorococcus* sp. MIT9301
Tery_0838	ATTTACCCctaATTAATGGaagGGTAAAAG	4.19E-05	−35	*Trichodesmium erythraeum* IMS101
SYNPCC7002_A2813	TTTACCTAtccATTAAGTAaaaGTTACCTG	7.16E-05	−124	*Synechococcus* sp. PCC7002
Tery_4465	GTAAATGTagtCTTAATTAttcCTCAAAAG	7.16E-05	−190	*Trichodesmium erythraeum* IMS101
Tery_4515	GTTAATCAttaCTTTTAAAgctCTTAACTT	7.16E-05	−432	*Trichodesmium erythraeum* IMS101
P9301_13641	CTTAACTTtccATTGGTAAagaATTAATAA	8.71E-05	−132	*Prochlorococcus* sp. MIT9301
Tery_1234	ATTAACTAtgtATTAATAAggaCTAGAATT	8.71E-05	−113	*Trichodesmium erythraeum* IMS101
PMT9312_1515	TTTATACCaatATTAATATgtaATTACTAG	8.95E-05	−30	*Prochlorococcus* sp. MIT9312
SYNPCC7002_G0011	TTTAAACCaatTTTAGATAagtTTTAGTTA	9.03E-05	−369	*Synechococcus* sp. PCC7002

Supplementary Table 3. Extracellular proteins of *Synechocystis* after four days of growth under P deficiency.

Protein index	Accession ID	Gene name	Description	Coverage (%)	Unique peptide	Abundance score
**1**	**P74649**	**sll0721**	**Leukotoxin LtxA**	**41**	**38**	**9.29E+09**
2	P72938	nucH	Uncharacterized protein YjiK	19	25	7.76E+09
3	P72939	sll0654	Alkaline phosphatase	37	37	7.48E+09
4	P73704	hofG	General secretion pathway protein G	40	6	5.37E+09
5	P73458	prc	Carboxyl-terminal protease	78	31	3.51E+09
6	Q55835	futA2	Iron uptake protein A2	62	18	2.91E+09
7	P74647	sll0723	Sll0723 protein	37	51	2.59E+09
8	P73019	frpC	Iron-regulated protein	21	11	1.48E+09
9	Q6ZEX5	slr5005	Slr5005 protein	40	102	1.39E+09
10	P73222	slr2005	Slr2005 protein	48	10	1.09E+09
11	P73785	pstS	Phosphate-binding protein	62	18	8.85E+08
12	P73590	slr1403	Slr1403 protein	20	36	8.8E+08
13	P73600	sll1785	Sll1785 protein	52	11	8.45E+08
14	P73139	slr1028	Slr1028 protein	16	39	7.25E+08
15	Q55365	slr0897	Endoglucanase	33	23	7.12E+08
16	P77961	glnA	Glutamine synthetase	41	15	6.56E+08
17	Q55549	slr0168	Slr0168 protein	35	14	6.29E+08
18	P73103	slr1908	Slr1908 protein	35	10	5.85E+08
19	P74789	sll0319	Sll0319 protein	72	12	5.73E+08
20	P72952	sll0645	Sll0645 protein	53	10	5.29E+08
21	P74109	sll1891	Sll1891 protein	41	6	5.25E+08
22	P73238	slr2018	Slr2018 protein	38	20	5.19E+08
23	P72923	slr1087	Slr1087 protein	29	7	4.88E+08
24	P73603	slr1852	Slr1852 protein OS	67	11	4.84E+08
25	Q55648	sll0314	Sll0314 protein	53	19	4.66E+08
26	P74500	slr1940	Slr1940 protein	37	13	4.64E+08
27	Q55547	sll0293	Sll0293 protein	19	3	4.22E+08
28	Q55847	sll0470	Sll0470 protein	35	8	4.18E+08
29	P74598	sll1491	Uncharacterized WD repeat-containing protein	33	9	4.17E+08
30	Q55426	slr0841	Slr0841 protein	51	9	3.81E+08
31	P73452	nrtA	Nitrate/nitrite binding protein NrtA	38	13	3.77E+08
32	P73595	slr1410	Uncharacterized WD repeat-containing protein	39	11	3.45E+08
33	Q55460	cmpA	Bicarbonate-binding protein CmpA	33	12	3.45E+08
34	P73107	sll1837	Sll1837 protein	46	6	3.39E+08
35	Q55199	pstS2	Phosphate-binding protein	34	9	3.3E+08
36	P74615	sll1483	Protein Sll1483	18	2	3.21E+08
37	P73126	sll0997	Probable thylakoid lumen protein Sll0997	51	12	3.04E+08
38	P54205	cbbL	Ribulose bisphosphate carboxylase large chain	36	19	2.79E+08
39	P73409	slr1841	Slr1841 protein	37	9	2.67E+08
40	Q55972	slr0708	Slr0708 protein	45	11	2.57E+08

Supplementary Table 4. Primers used in this study.

Primers	Sequences (5'-3')	Usage [a]
sll0217-RT-1	5'-ATGGTTACCCTAATTGATTCTCCAACCTCTGC-3'	a
sll0217-RT-2	5'-AACTTGCGATGGGACGTGTCGATGAG-3'	a
sll0219-RT-1	5'-CTCAAACCAAAGTGTTACGGTCTCGCC-3'	a
sll0219-RT-2	5'-CGCAGTAATTGCTCCAGGGTAACCATGC-3'	a
sll0221-RT-1	5'-GGATTGGCAGACTTACCCACCTATGCC-3'	a
sll0221-RT-2	5'-ACCTGGGCATTGAGAGCTTGATATTTGGTC-3'	a
slr0242-RT-1	5'-CCACTGCCTTAGAAACTAATCAACCAGCC-3'	a
slr0242-RT-2	5'-AATCCGGACTAACACCGACAACAACAGC-3'	a
sll0247-RT-1	5'-GTGCAAACCTATGGCAACGACACCG-3'	a
sll0247-RT-2	5'-GTAGGGATAGGTATCCACAATTTGTCCGC-3'	a
sll0550-RT-1	5'-CTGCTATCCGTTCTTTGGACTGGGACC-3'	a
sll0550-RT-2	5'-TTACTAACCCACTGTGGTCTGGCTCAG-3'	a
sll0680-RT-1	5'-TTGTCCAAGCATTTAGTGCCCACAGC-3'	a
sll0680-RT-2	5'-GCTTGATTGAGGGCCACAAACCAGC-3'	a
sll0755-RT-1	5'-ATGACAGAGGTATTAAGGGTAGGACAGCC-3'	a
sll0755-RT-2	5'-TGTGAAAATTCGCTGTCTACGGAGATGCC-3'	a
sll0720-RT-1	5'-TCAATGAAAACGCTCTAAAAATACTGGGCGA-3'	a
sll0720-RT-2	5'-GATCATTAATAAAAGCCCAACAAGCAAAGCCA-3'	a
sll0721-RT-1	5'-GAGGGTTTCAAAATGGGGAAAATATGGCA-3'	a
sll0721-RT-2	5'-CGTGGTGGCCGTAGATGACATCGT-3'	a
slr1171-RT-1	5'-TGCCCAAGCCAACAACACAATCTACGG-3'	a
slr1171-RT-2	5'-CAAACTGATTACAGGGAAAACCAAGCACCG-3'	a
slr1198-RT-1	5'-GACTTTACCCAAGAATCCAGCCAAGGC-3'	a
slr1198-RT-2	5'-TGGGTTTCGTCAATATCACAAATCCAGCC-3'	a
slr1247-RT-1	5'-AGTTGCTACCTTCTCCGTAGTCAGCG-3'	a
slr1247-RT-2	5'-TGGCACCAAAATCAACGGTTTCACCG-3'	a
sll1292-RT-1	5'-AGTTGTGGAAGATACCAAGTCTGACCAGT-3'	a
sll1292-RT-2	5'-ATCTTCTAGGGCCAATTCTCCCCGA-3'	a
sll1294-RT-1	5'-GGTTAGCACCCCCACTAAACCCCA-3'	a
sll1294-RT-2	5'-GCTAAAGCCTTACTGATGGTTTCGCCA-3'	a
sll1296-RT-1	5'-ATGTTTGATGCCGCCACCCTCGC-3'	a
sll1296-RT-2	5'-GGGGTTAAAGCCGACATTCCCGCT-3'	a
sll1468-RT-1	5'-CCAGGAGTCCGTCATAGTAATGCAGGC-3'	a
sll1468-RT-2	5'-CCAGCACCGAACAACTAAAACAAAGCCAG-3'	a
slr1516-RT-1	5'-ACTTACCTTACGACTACACCGCTCTGG-3'	a
slr1516-RT-2	5'-GCAATTCCAGTAGAAACTATGGTTCCAAGC-3'	a
sll1521-RT-1	5'-ATGCCTGCCAAAGACGTTCAAATCTGC-3'	a
sll1521-RT-2	5'-CGTAATCCAGGCTGTTGAGGTCTAACCG-3'	a
sll1621-RT-1	5'-GTGTCCGTGACGAATCTGTACCTGGTC-3'	a
sll1621-RT-2	5'-TGTTTGCCCCATTGGAACATAACAAAGGC-3'	a
slr1651-RT-1	5'-GGTGTAAAAAATCGCATTACTTGCATGGCT-3'	a
slr1651-RT-2	5'-TGCCGGTAAAGCCCACTCATCCAA-3'	a

slr1894-RT-1	5'-ATATCGGCATTCCTGAAGCGGACCG-3'	a
slr1894-RT-2	5'-GGATACGCTCAGCAATGTCATCCACC-3'	a
sll1987-RT-1	5'-CCAGTAAATGTCCTGTAATGCACGGAGC-3'	a
sll1987-RT-2	5'-CAACTTTGGCTATCGGTCATCAAATGGTG-3'	a
slr1908-RT-1	5'-TAGCTCCTTGGCTATTGTTCCCGGC-3'	a
slr1908-RT-2	5'-GCACATCCCGCAACTCGGACACA-3'	a
slr1992-RT-1	5'-CGGAGGTCATTGCCGATAAAGTTGTCC-3'	a
slr1992-RT-2	5'-GTGAAATCAACGTCGTACTTGGTTTTGGTG-3'	a
ssl2559-RT-1	5'-TTTTTGCCCCTATCACTAGAATTTAACGCTTG-3'	a
ssl2559-RT-2	5'-TGAACATCATCAGGAGCCAGAATAGCCA-3'	a
rnpB-RT-1	5'-GAGTTGCGGATTCCTGTCA-3'	a
rnpB-RT-2	5'-ACTGCTGGTGCGCTCTTAC-3'	a
sll1341-Y1H-1	5'-atcGGTACCAAGTAAACAGCCGTGG-3'	b
sll1341-Y1H-2	5'-gatCTCGAGTGGTCTATCCACGGTG-3'	b
slr1516-Y1H-1	5'-agaCTCGAGGAGAAGGTTGCTTCGA-3'	b
slr1516-Y1H-2	5'-ccgGGTACCTTTTAGCTTTTCCTCACA-3'	b
slr1890-Y1H-1	5'-gcgGGTACCGACAAACAAATCAGCC-3'	b
slr1890-Y1H-2	5'-tatCTCGAGTTCACGGTCCGACGAC-3'	b
slr1908-Y1H-1	5'-ccgCTCGAGGTTTATTTGCTTGCCTG-3'	b
slr1908-Y1H-2	5'-tctGGTACCAACAAAGGGATCCTCACA-3'	b
sll1291-EMSA-1	5'-ACTAGGTCTTGAATTGCCCCATCGCT-3'	c
sll1291-EMSA-2	5'-GATTGCATGGGACAACAATGGCGGT-3'	c
slr1516-EMSA-1	5'-GCAAGTAAAGACCAGGCCATTGTCGA-3'	c
slr1516-EMSA-2	5'-GGTGTAGTCGTAAGGTAAGTTAGGTAGTGCG-3'	c
slr1651-EMSA-1	5'-CAGCAGATGAGACTGATAGGCTGGGTA-3'	c
slr1651-EMSA-2	5'-AGCCATGCAAGTAATGCGATTTTTTACACCA-3'	c
slr1908-EMSA-1	5'-GTTTATTTGCTTGCCTGTCAATGGAAATGATGATG-3'	c
slr1908-EMSA-2	5'-AACAAAGGGATCCTCACACCGATTACATTAATC-3'	c
slr0081-KO-1	5'-GGTTAAATACCATCTAGTAGGTTTCCCAATGGC-3'	d
slr0081-KO-2	5'-GCTAAAGGGCTTGATATCAGTGATGTAGTCATCGGC-3'	d
slr0081-KO-3	5'-GACTACATCACTGATATCAAGCCCTTTAGCCTGAAG-3'	d
slr0081-KO-4	5'-AACGATAGCCAAAGCCCCGCACTGT-3'	d
slr1516-OE-1	5'-ATGGCTTACGCACTACCTAACTTACCTTACGAC-3'	d
slr1516-OE-2	5'-CTAGGCCGCTGCTAAGTTAGCCGCC-3'	d
sll0337^{T214N}-1	5'-ATGGAAATAATTACATTGGCGATCGGCGG-3'	d
sll0337^{T214N}-2	5'-AGCCACCGCATTTAAAGGGGTTCGTAACTCG-3'	d
sll0337^{T214N}-3	5'-GAACCCCTTTAAATGCGGTGGCTTTGATTGCGG-3'	d
sll0337^{T214N}-4	5'-CTAGGGATTTTCATCCATATTCGGTTCATAGGG-3'	d
slr1908-OE-1	5'-ATGAATAAGTTGACCAGTCATTTACTGAAATTATTTCCG-3'	d
slr1908-OE-2	5'-CTAGAACTTGAAGGTGGTGCGGAGTACACCA-3'	d
slr2031-P1	5'-GTGGTCATTCTCAAGGAGTTGGTGGCTAAGTTGTACCG-3'	d
slr2031-P2	5'-TGATCACGCGTGCTAGCGGGTGTTATAACAGGCGATC-3'	d
slr2031-P3	5'-CACCCGCTAGCACGCGTGATCAAACCGCTGTGGCG-3'	d
slr2031-P4	5'-CCCGCAGAACGACCAATGTCATATCGTCTCGGGC-3'	d

slr0081-Flag$_3$-1	5’-CCAAAGGCAGTGAAATGGAAAAAGTAACTGG-3’	d
slr0081-Flag$_3$-2	5’−GATATCCTACTTATCGTCGTCATCCTTGTAATCGATCTTATCGTC GTCATCCTTGTAATCTCCCTTATCGTCGTCATCCTTGTAATCACCA AAACGATAGCCAAAGCC-3’	d
slr0081-Flag$_3$-3	5’−GACGACGATAAGTAGGATATCAAAATTATTATTTATTTAAACTT TTTTGCTCAGAACCTC-3’	d
slr0081-Flag$_3$-4	5’-GACGTAATCTGCTAATTCTTCATTGGCATCGAC-3’	d
slr1908-His$_8$-1	5’-CGCATTGCTAGTAATCCCTTTTACCAGGC-3’	d
slr1908-His$_8$-2	5’−GATATCCTAGTGGTGATGATGATGATGATGATGGAACTTGAAGG[illegible] TGCG-3’	d
slr1908-His$_8$-3	5’-CATCACCACTAGGATATCGAACTTTGTTGGGGTAAA-3’	d
slr1908-His$_8$-4	5’-GATGAAGGCGTAGCCATCCATCTCCG-3’	d
slr0081-exp-1	5’-GGCGAATTCATGTTGGTTAAATACCATCTAGTAGG-3’	e
slr0081-exp-2	5’-TGTCTCGAGCTAACCAAAACGATAGCCA-3’	e

[a] a, used for qRT-PCR; b, used for yeast one-hybrid assay; c, used for electrophoretic mobility shift assay; d, used for construction of mutants; e, used for recombinant protein production.

Supplementary Table 5. Strains and plasmids used in this study.

Strains and plasmids	Derivation and/or relevant characteristics [a]	Reference or source
Synechocystis		
Synechocystis sp. PCC 6803	Wild type	Pasteur Culture Collection
Δ*phoB*	Km^r, *Synechocystis* 6803 mutant, the result of transformation with pHS10464	This study
PhoB-$Flag_3$	Km^r, *Synechocystis* 6803 mutant, the result of transformation with pHS10925	This study
$PhoR^{T214N}$	Sp^r, Synechocystis 6803 mutant, the result of transformation with pHS14866	This study
SodB-OE	Sp^r, Synechocystis 6803 mutant, the result of transformation with pHS10750	This study
Slr1908-OE	Km^r, Synechocystis 6803 mutant, the result of transformation with pHS14939	This study
Slr1908-His_8	Km^r, Synechocystis 6803 mutant, the result of transformation with pHS10358	This study
*slr1908*KD	Sp^r, *Synechocystis* 6803 mutant, the result of transformation with pHS5759	[1]
**Yeast**		
Y1HGold	MATα, ura3-52, his3-200, ade2-101, trp1-901, leu2-3, 112, gal4Δ, gal80Δ, met–, MEL1	Clontech
Y1HGold(*pro-sodB*::PGAD)	Ap^r, Y1HGold transformed with pHS10216 and PGADT7	This study
Y1HGold(*pro-sodB*::PhoB)	Ap^r, Y1HGold transformed with pHS10216 and pHS8307	This study
Y1HGold(*pro-sodB*::RpaB)	Ap^r, Y1HGold transformed with pHS10216 and pHS8311	This study
Y1HGold(*pro-slr1908*::PGAD)	Ap^r, Y1HGold transformed with pHS10214 and PGADT7	This study
Y1HGold(*pro-slr1908*::PhoB)	Ap^r, Y1HGold transformed with pHS10214 and pHS8307	This study
Y1HGold(*pro-bfrA*::PGAD)	Ap^r, Y1HGold transformed with pHS13032 and PGADT7	This study
Y1HGold(*pro-bfrA*::PhoB)	Ap^r, Y1HGold transformed with pHS13032 and pHS8307	This study
Y1HGold(*pro-bfrB*::PGAD)	Ap^r, Y1HGold transformed with pHS13036 and PGADT7	This study
Y1HGold(*pro- bfrB*::PhoB)	Ap^r, Y1HGold transformed with pHS13036 and pHS8307	This study
**Plasmids**		
pHS298	Ap^r Sp^r, P*psbAII* expression vector	[2]
pHS2601	Ap^r, PCR fragment containing the upstream sequence of slr1908 cloned into pMD18T vector	[1]
pHS3706	Ap^r, PCR fragment containing the downstream sequence of slr1908 cloned into pMD18T vector	[1]
pHS3823	Ap^r Sp^r, fragment Omega–P*petE* from pHS185 which was excised by Sal I/BamH I was inserted into the PstI site of pHS3706	[1]
pHS5759	Ap^r Sp^r, fragment Omega–P*petE-slr1908D* from pHS3823 which was excised by Hind III/Xba I was inserted into the Pst I site of pHS2601	[1]
pHS8307	Ap^r, PCR fragment containing *slr0081* cloned into PGADT7 vector	This study
pHS8311	Ap^r, PCR fragment containing *slr0947* cloned into PGADT7 vector	This study
pHS10216	Ap^r, PCR fragment containing *slr1516* promoter cloned into pAbAi vector	This study
pHS10214	Ap^r, PCR fragment containing *slr1908* promoter cloned into pAbAi vector	This study
pHS10358	Ap^r Km^r, PCR fragment containing *slr1908*-His_8	This study

	cloned into pMD18T vector, and a C.K2 cassette from pRL446 was inserted into the EcoRV site	
pHS10433	Ap^r, PCR fragment containing *slr0081* cloned into pMD18T vector	This study
pHS10464	Ap^r Km^r, C.K2 cassette from pRL446 inserted into pHS10433 at the EcoRV site	This study
pHS10750	Ap^r Sp^r, PCR fragment containing *slr1516* inserted into pHS298 at the NdeI site	This study
pHS10902	Ap^r, PCR fragment containing *slr0081*-$Flag_3$ cloned into pMD18T vector	This study
pHS10925	Ap^r Km^r, C.K2 cassette from pRL446 inserted into pHS10902 at the EcoRV site	This study
pHS11035	Ap^r Sp^r, PCR fragment containing *slr0081* inserted into pHS298 at the NdeI site	This study
pHS13032	Ap^r, PCR fragment containing *sll1341* promoter cloned into pAbAi vector	This study
pHS13036	Ap^r, PCR fragment containing *slr1890* promoter cloned into pAbAi vector	This study
pHS14368	Ap^r, PCR fragment containing *slr2031* cloned into pMD18T vector	This study
pHS14370	Ap^r Km^r, C.K2 cassette from pRL446 inserted into pHS14368 at the BamHI site	This study
pHS14838	Km^r, PCR fragment containing *slr0081* cloned into pET41a vector	This study
pHS14866	Ap^r Sp^r, PCR fragment containing $sll0337^{T214N}$ inserted into pHS298 at the NdeI site	This study
pHS14939	Ap^r Km^r, fragment containing P*psbAII-slr1908* was inserted into pHS14370 at the MluI site	This study
pGADT7	Ap^r, yeast two-hybrid expression vector with ADH1 promoter and a fusion of GAL4 AD	Clontech
pRL446	Km^r, a cloning vector with a kanamycin resistance marker (C.K2)	[3]

[a] Ap, ampicillin; Km, kanamycin; Sp, spectinomycin.

Supplementary References

1. Qiu, G. W. *et al.* A unique porin meditates iron-selective transport through cyanobacterial outer membranes. *Environ. Microbiol.* **23**, 376–390 (2021).
2. Jiang, H. B. *et al.* Sll1263, a unique cation diffusion facilitator protein that promotes iron uptake in the cyanobacterium *Synechocystis* sp. Strain PCC 6803. *Plant Cell Physiol.* **53**, 1404–1417 (2012).
3. Elhai, J. & Wolk, C. A versatile class of positive-selection vectors based on the nonviability of palindrome-containing plasmids that allows cloning into long polylinkers. *Gene* **68**, 119–138 (1988).
