# Supplementary material for: Phosphorus deficiency alleviates iron limitation in Synechocystis cyanobacteria through direct PhoB-mediated gene regulation
Source: Nat Commun. 2024 May 24;15:4426. doi: 10.1038/s41467-024-48847-4 (PMC11126600; doi:10.1038/s41467-024-48847-4)
Supplement: Supplementary file 3 — Description of Additional Supplementary Files [file 41467_2024_48847_MOESM3_ESM.pdf]

Description of Additional Supplementary Files:

Supplementary Data 1: List of the putative Pho regulons. Genes studied or discussed in this study are shown in bold red font.

Supplementary Data 2: The transcriptional responses of Synechocystis under different Fe and P availabilities.

Supplementary Data 3: List of the differentially expressed genes specific to −Fe−P.
