# Supplementary material for: Phosphorus deficiency alleviates iron limitation in Synechocystis cyanobacteria through direct PhoB-mediated gene regulation
Source: Nat Commun. 2024 May 24;15:4426. doi: 10.1038/s41467-024-48847-4 (PMC11126600; doi:10.1038/s41467-024-48847-4)
Supplement: Supplementary file 7 — Reporting Summary [file 41467_2024_48847_MOESM7_ESM.pdf]

nature portfolio

Corresponding author(s): Bao-Sheng Qiu

Last updated by author(s): May 3, 2024

Reporting Summary

Nature Portfolio wishes to improve the reproducibility of the work that we publish. This form provides structure for consistency and transparency in reporting. For further information on Nature Portfolio policies, see our Editorial Policies and the Editorial Policy Checklist.

Statistics

For all statistical analyses, confirm that the following items are present in the figure legend, table legend, main text, or Methods section.

n/a	Confirmed	
[ ]	[x]	The exact sample size (*n*) for each experimental group/condition, given as a discrete number and unit of measurement
[ ]	[x]	A statement on whether measurements were taken from distinct samples or whether the same sample was measured repeatedly
[ ]	[x]	The statistical test(s) used AND whether they are one- or two-sided<br>*Only common tests should be described solely by name; describe more complex techniques in the Methods section.*
[x]	[ ]	A description of all covariates tested
[x]	[ ]	A description of any assumptions or corrections, such as tests of normality and adjustment for multiple comparisons
[ ]	[x]	A full description of the statistical parameters including central tendency (e.g. means) or other basic estimates (e.g. regression coefficient) AND variation (e.g. standard deviation) or associated estimates of uncertainty (e.g. confidence intervals)
[ ]	[x]	For null hypothesis testing, the test statistic (e.g. *F*, *t*, *r*) with confidence intervals, effect sizes, degrees of freedom and *P* value noted<br>*Give P values as exact values whenever suitable.*
[x]	[ ]	For Bayesian analysis, information on the choice of priors and Markov chain Monte Carlo settings
[x]	[ ]	For hierarchical and complex designs, identification of the appropriate level for tests and full reporting of outcomes
[x]	[ ]	Estimates of effect sizes (e.g. Cohen's *d*, Pearson's *r*), indicating how they were calculated

Our web collection on statistics for biologists contains articles on many of the points above.

Software and code

Policy information about availability of computer code

Data collection	Flow cytometry data acquisition: BD FACSVerse<br>Absorption spectra data acquisition: Specord 210 plus spectrophotometer<br>Chlorophyll content determination: Shimadzu UV-2700 spectrophotometer<br>Image collection for cell size determination: Olympus BX50 microscopy<br>Measurement of chlorophyll fluorescence: WATER-PAM fluorometer (Walz GmbH, Germany)<br>qRT-PCR: 7900HT Fast real-time PCR system (Thermo Fisher, USA)<br>ROS content determination: F-4500 fluorescence spectrophotometer (Hitachi, Japan)<br>Fe content quantification: inductively coupled plasma mass spectrometry (ICP-MS, Thermos Scientific)<br>RNA sequencing: Illumina HiSeq×TEN sequencer<br>ChIP sequencing: Illumina NovaSeq 6000<br>LC-MS/MS spectra: Q-Exactive Plus Orbitrap mass spectrometer (Thermo Fisher).

Data analysis	Flow cytometry data analysis: FlowJo_v10.8.1<br>Cell size determination and immunoblotting quantification: ImageJ-win64<br>Statistical analyses: Origin 2021 software<br>ChIP-seq data analysis: Trimmomatic (version 0.36), Bwa (version 0.7.15), Samtools (version 1.3.1), MACS2 software (version 2.1.1.20160309)<br>Transcriptomic data analysis: Majorbio Cloud Platform (www.majorbio.com);<br>LC–MS/MS data analysis: Proteome Discoverer 2.1.

For manuscripts utilizing custom algorithms or software that are central to the research but not yet described in published literature, software must be made available to editors and reviewers. We strongly encourage code deposition in a community repository (e.g. GitHub). See the Nature Portfolio guidelines for submitting code & software for further information.

Data

Policy information about availability of data

All manuscripts must include a data availability statement. This statement should provide the following information, where applicable:

- Accession codes, unique identifiers, or web links for publicly available datasets
- A description of any restrictions on data availability
- For clinical datasets or third party data, please ensure that the statement adheres to our policy

Data supporting the findings of this work are available in the Supplementary Data and Source Data files. The transcriptomic data generated in this study have been deposited in NCBI SRA database under the accession code PRJNA1080180 [https://www.ncbi.nlm.nih.gov/bioproject/PRJNA1080180]. The ChIP-Seq data have been deposited in NCBI GEO database under the accession code GSE260812 [https://www.ncbi.nlm.nih.gov/geo/query/acc.cgi?acc=GSE260812]. The proteomics data have been deposited to the ProteomeXchange Consortium under accession code PXD050522 [https://www.ebi.ac.uk/pride/archive/projects/PXD050522/private]. TARA metagenomes and metatranscriptomes were analyzed using the Ocean Gene Atlas portal [https://tara-oceans.mio.osupytheas.fr/ocean-gene-atlas/], and the data generated in this study were provided in the Source Data file. Source data are provided with this paper.

Research involving human participants, their data, or biological material

Policy information about studies with human participants or human data. See also policy information about sex, gender (identity/presentation), and sexual orientation and race, ethnicity and racism.

Reporting on sex and gender	Not applicable for this study.
Reporting on race, ethnicity, or other socially relevant groupings	Not applicable for this study.
Population characteristics	Not applicable for this study.
Recruitment	Not applicable for this study.
Ethics oversight	Not applicable for this study.

Note that full information on the approval of the study protocol must also be provided in the manuscript.

Field-specific reporting

Please select the one below that is the best fit for your research. If you are not sure, read the appropriate sections before making your selection.

☒ Life sciences ☐ Behavioural & social sciences ☐ Ecological, evolutionary & environmental sciences

For a reference copy of the document with all sections, see nature.com/documents/nr-reporting-summary-flat.pdf

Life sciences study design

All studies must disclose on these points even when the disclosure is negative.

Sample size	Sample sizes were determined based on the generation of convincing and compelling results. The sample size was not predetermined using statistical methods. Unless noted otherwise, all experiments were carried out with at least three independent biological replicates. Similar results were obtained from the replicates of each experimental analysis.
Data exclusions	No data were excluded from the analysis.
Replication	All experiments were repeated independently from 2 to 6 times, and the number of independent experiments or biological replicates is indicated in the figure legends.
Randomization	Samples were allocated randomly into experimental groups.
Blinding	The investigators were blinded to group allocation during data collection and analysis.

Reporting for specific materials, systems and methods

We require information from authors about some types of materials, experimental systems and methods used in many studies. Here, indicate whether each material, system or method listed is relevant to your study. If you are not sure if a list item applies to your research, read the appropriate section before selecting a response.

Materials & experimental systems

n/a	Involved in the study	
☐	☒	Antibodies
☒	☐	Eukaryotic cell lines
☒	☐	Palaeontology and archaeology
☒	☐	Animals and other organisms
☒	☐	Clinical data
☒	☐	Dual use research of concern
☒	☐	Plants

Methods

n/a	Involved in the study	
☐	☒	ChIP-seq
☐	☒	Flow cytometry
☒	☐	MRI-based neuroimaging

Antibodies

Antibodies used

Anti-Flag: Mouse antibody; Supplier name: Sigma; Product number: F1804; Lot number: SLBJ4607V; Dilution: 1:1000.
Anti-His: Mouse antibody; Supplier name: Proteintech; Catalog number: 66005-1-Ig; Lot number:10020245; Dilution: 1:1000.
Anti-D1: Rabbit antibody; Supplier name: PhytoAB; Catalog number: PHY5272S; Lot number:2725A5; Dilution: 1:1000.
Anti-PsaD: Rabbit antibody; from Guo-Zheng Dai et al., DOI: 10.1093/plcell/koad330, Dilution: 1:2000.
Alkaline Phosphatase-conjugated Goat Anti-Mouse IgG(H+L) antibody, Supplier name: Proteintech; Catalog number: SA00002-1; Lot number:20000425; Dilution: 1:2000.
Alkaline Phosphatase-conjugated Goat Anti-Rabbit IgG(H+L) antibody, Supplier name: Proteintech; Catalog number: SA00002-2; Lot number:20000815; Dilution: 1:2000.

Validation

The D1 antibody purchased from PhytoAB was validated by the company and our previous study (Guo-Zheng Dai et al., DOI: 10.1093/plcell/koad330). The PsaD antibody was confirmed by our previous study (Guo-Zheng Dai et al., DOI: 10.1093/plcell/koad330). The specific reorganization of Anti-Flag and Anti-His to tagged proteins was confirmed by us in this study and by the validation statements on manufacturer's website:https://www.sigmaaldrich.cn/CN/zh/product/sigma/f1804, https://www.ptgcn.com/products/His-Tag-Antibody-66005-1-Ig.htm.

Plants

Seed stocks

Not applicable for this study.

Novel plant genotypes

Not applicable for this study.

Authentication

Not applicable for this study.

ChIP-seq

Data deposition

☒ Confirm that both raw and final processed data have been deposited in a public database such as GEO.

☒ Confirm that you have deposited or provided access to graph files (e.g. BED files) for the called peaks.

Data access links
May remain private before publication.

https://www.ncbi.nlm.nih.gov/geo/query/acc.cgi?acc=GSE260812

Files in database submission

Sample file:
IN_PhoB_Flag_1_1.fq.gz
IN_PhoB_Flag_1_2.fq.gz
IN_PhoB_Flag_2_1.fq.gz
IN_PhoB_Flag_2_2.fq.gz
IP_PhoB_Flag_1_1.fq.gz
IP_PhoB_Flag_1_2.fq.gz

IP_PhoB_Flag_2_1.fq.gz
IP_PhoB_Flag_2_2.fq.gz
PhoB_Flag.peak_annotation.xls
IN_PhoB_Flag_1.tdf
IN_PhoB_Flag_2.tdf
IP_PhoB_Flag_1.tdf
IP_PhoB_Flag_2.tdf
IP_PhoB_Flag_1_vs_IN_PhoB_Flag_1_peaks.xls.bed
IP_PhoB_Flag_2_vs_IN_PhoB_Flag_2_peaks.xls.bed
seq_template.xlsx
IN_PhoB_Flag_1_1.fq.gz.MD5
IN_PhoB_Flag_1_2.fq.gz.MD5
IN_PhoB_Flag_2_1.fq.gz.MD5
IN_PhoB_Flag_2_2.fq.gz.MD5
IP_PhoB_Flag_1_1.fq.gz.MD5
IP_PhoB_Flag_1_2.fq.gz.MD5
IP_PhoB_Flag_2_1.fq.gz.MD5
IP_PhoB_Flag_2_2.fq.gz.MD5
PhoB_Flag.peak_annotation.xls.MD5

Genome browser session (e.g. UCSC): None

Methodology

Replicates	Two biological replicates with high overlap for ChIP-seq peaks.
Sequencing depth	For IP_PhoB_Flag_1, 36605702 total reads, 27884956 mapped reads, 27180238 paired, and 58606 single-end.<br>For IP_PhoB_Flag_2, 35610010 total reads, 26745232 mapped reads, 26188514 paired, and 49404 single-end.
Antibodies	ANTI-FLAG monoclonal antibody produced in mouse (Sigma, F1804)
Peak calling parameters	MACS2 software (version 2.1.1.20160309) was used to call peaks by default parameters (bandwidth,300 bp; model fold, 5, 50;q value, 0.05).
Data quality	The samples from two biological replicates show highly consistent results, with 24 peaks enriched over 5-fold.
Software	Low-quality reads were filtered via Trimmomatic (version 0.36) software. The clean reads were mapped to the genome by Bwa (version 0.7.15). Samtools (version 1.3.1) was used to remove potential PCR duplicates. And MACS2 software (version 2.1.1.20160309) was used to call peaks by default parameters (bandwidth,300 bp; model fold, 5, 50; q value, 0.05).

Flow Cytometry

Plots

Confirm that:

- [x] The axis labels state the marker and fluorochrome used (e.g. CD4-FITC).
- [x] The axis scales are clearly visible. Include numbers along axes only for bottom left plot of group (a 'group' is an analysis of identical markers).
- [x] All plots are contour plots with outliers or pseudocolor plots.
- [x] A numerical value for number of cells or percentage (with statistics) is provided.

Methodology

Sample preparation	At each time point, 1 mL cells were collected and fixed with glutaraldehyde (Sigma-Aldrich) at a final concentration of 0.2%. The fixed samples were frozen and stored at −80°C
Instrument	BD FACSVerse
Software	Flow cytometry: BD FCASuite_v1.0.6<br>Data analysis: FlowJo_v10.8.1
Cell population abundance	Data analyses were performed using at least 10,000 cells in each analysis.
Gating strategy	The gating was performed as described in the main text. The Auto-fluorescence signal (PerCP-Cy5.5) was detected at the excitation wavelength of 488 nm to reflect the chlorophyll content by fluorescence intensity.

- [x] Tick this box to confirm that a figure exemplifying the gating strategy is provided in the Supplementary Information.
