# Supplementary material for: Phosphorus deficiency alleviates iron limitation in Synechocystis cyanobacteria through direct PhoB-mediated gene regulation
Source: Nat Commun. 2024 May 24;15:4426. doi: 10.1038/s41467-024-48847-4 (PMC11126600; doi:10.1038/s41467-024-48847-4)
Supplement: Supplementary file 2 — Peer Review File [file 41467_2024_48847_MOESM2_ESM.pdf]

nature portfolio

Peer Review File

Phosphorus deficiency alleviates iron limitation in Synechocystis cyanobacteria through direct PhoB-mediated gene regulation

Open Access This file is licensed under a Creative Commons Attribution 4.0 International License, which permits use, sharing, adaptation, distribution and reproduction in any medium or format, as long as you give appropriate credit to the original author(s) and the source, provide a link to the Creative Commons license, and indicate if changes were made. In the cases where the authors are anonymous, such as is the case for the reports of anonymous peer reviewers, author attribution should be to 'Anonymous Referee' followed by a clear attribution to the source work. The images or other third party material in this file are included in the article's Creative Commons license, unless indicated otherwise in a credit line to the material. If material is not included in the article's Creative Commons license and your intended use is not permitted by statutory regulation or exceeds the permitted use, you will need to obtain permission directly from the copyright holder. To view a copy of this license, visit http://creativecommons.org/licenses/by/4.0/.

Reviewer #1 (Remarks to the Author):

This work deals with the interplay between regulatory mechanisms relating responses to deficiency of various elements required for growth, namely Fe and P, in cyanobacteria. This condition is frequently found in natural environments. The authors point to the interest of the knowledge of mechanism relating P and Fe limitation in oceanic planktonic cyanobacteria; however, the present study has been performed on the model strain Synechocystis sp. PCC 6803, whose biology considerably differ from that of marine strains. It would have been interesting to study the mechanisms of adaptation to P/Fe limitation in Prochorococcus or marine Synechococcus. The data presented in Fig. 3 are rather indirect for this purpose. An important issue: the work presented needs improved (or clarified) statistic treatment. At first glance, some of the differences taken into account look of low statistical significance being the results of statistic tests rather unclear (see below).

-Page 4, for comparison of growth under different conditions, an estimation of growth rate constant (based on total protein or chlorophyll determination, or even OD) would be more adequate than cell number (also for data on Fig. 6D and 7A). Statistic comparison of data on photosynthetic activity and cell diameter presented in Fig. 1 is not clear. How many cells from how many cultures have been used for data in Fig. 1E? What is the p value corresponding to a, b and c in Figs. 1D and 1E? The same apply for Fig. 2A, 2B and 2D, Fig. 3A, Fig. 5C, 6C.

-Fig. 4. Data on Fig. 4A and 4B (in the absence and presence of MV) appears similar; thus, the sentence on lines 131-133 might not be sustained. Also, these results question the relationship between ETR and MV and superoxide levels (lines 138-140). (Again, statistical comparisons would be very informative here.)

-The role of PhoB on the regulation of sodB. In the phoB mutant, the expression of sodB in the absence of P is diminished with regard to the WT; however, in the mutant the levels are still higher in the absence than in the presence of P. Thus, PhoB could act as an enhancer of sodB expression, but other factor(s) would participate in the response to Fe/P availability. Please, comment on this. Please, label the different flasks in Fig. 5E.

-The first paragraphs of the Discussion section deal with the connections of cellular adaptations under Fe and P limitations, including a higher requirement for Fe under conditions of P limitation (line 232-233) and the phenomenon of increased Fe content under P deficiency (lines 239-240). According to this, it would be expected that growth was higher under conditions of -P/+Fe than of -P/-Fe. It would be nice to include data on the -P/+Fe condition in the growth experiment of Fig. 1A.

-Lines 288-289. Please change "universal role" to "widespread role".

Reviewer #2 (Remarks to the Author):

Qiu et al., show that the cyanobacterium Synechocytis PCC6803 grows better at combined Fe/P limitation than at Fe repletion alone. Furthermore, the authors show that the effect is lost in a ΔphoB strain. The PhoB regulon was investigated using ChIP-seq. Specifically sodB, slr1908 coding for an iron-selective porin and several genes involved in cyclic electron flow were identified to be directly positively regulated by PhoB. Finally, the authors propose that the improved growth at double depletion conditions is caused by maintaining intracellular ROS homeostasis, by enhancing Fe uptake capacity and by increasing cyclic electron flow.
These are interesting results that confirm the counterintuitive findings in other studies. I do not doubt the general effect and the data regarding the PhoB regulon, but I am not yet convinced by the interpretation of the results in terms of the -Fe+P and -Fe-P phenotypes. Several aspects remain puzzling to me. Those should be addressed prior to publication in a high-ranking journal.

Major concerns:
1. Despite being driven by previous data and the ChIP-seq results, the focus on sodB, slr1908 and the CES genes as main contributors to the observed phenotype seems too narrow to me. I would rather recommend transcriptome analysis at -Fe and -P vs +P+Fe alone to cover the baseline transcriptional response at these conditions in order to compare it with the -Fe-P response to cover all transcriptional adaptions. In the current approach important changes might be missed.
2. The general iron stress response by Fur/Isar1 is completely neglected in the data interpretation and discussion.
a. The majority of the transcripts that are discussed to cause the improved growth at -Fe-P (sodB, petF, Cytb6f) are actively repressed at -Fe through IsaR1. Actually, sodB is the textbook example of a gene that is repressed at iron limitation in a wide range of organism (E. coli, S aureus, Shewanella ...) including PCC6803. The ferrodoxin (petF) is e.g. replaced by a copper dependent alternative plastocyanin (petE) to spare iron. This indicates that it is in general a fitness advantage to repress these genes under iron limitation. It is not obvious to me why this regulation should have survived evolution if, in fact, it is the reason for a fitness disadvantage.
b. All iron transporters should be strongly up-regulated by the Fur regulon at -Fe. I would assume that the cells already max their Fe uptake capacity at iron limitation alone. Slr1908 might not be Fur dependent, but transcriptome data from PCC6803 show that it is induced at -Fe and -P conditions in a similar strength (Kopf et al 2014). Also, the cited paper (Qiu et al 2021) states that "the expression of slr1908 was significantly upregulated in Fe-deplete medium". Even if the levels are further increased at -Fe-P in addition to all the other iron up-take systems, it needs to be shown that this actually increases the intracellular iron concentrations. Slr1908 seems to be a major iron transporter and even be relevant at iron replete conditions: " slr1908 is highly expressed also under Fe-replete conditions. When grown in iron-sufficient medium, the slr1908KD strain exhibits a range of phenotypes that, taken together, are clearly indicative of iron limitation: lowered intracellular Fe quotas, induction of isiA and lowered PSI content and activity" (Qiu 2021). Thus, it seems not surprising to me that the slr1908kd does very bad at -Fe and can not be recovered by -P.
3. Together the above points leave me with two possible explanations:
a. If the interpretation of the authors is correct, the iron stress regulation of PCC6803 is sub-optimal at -Fe+P conditions. PhoB could lead to a re-allocation of the sparse iron from other iron dependent proteins to CES and sodB. That could be potentially seen in transcriptome data. However, are -Fe+P conditions so rare in natural environments that there is no selective pressure to optimize the iron stress regulation during evolution? Is there anything known of the Fe and P concentrations at diverse environments and seasons?
b. In an alternative potential explanation, the organisms do the best possible adaption to the -Fe+P conditions and the high P levels are for some reason detrimental by themself if the cells are iron depleted. In this interpretation the cells are not able to keep the optimal ratios (e.g. Redfield ratio) at -Fe+P and hence the lowered phosphate levels alone rebalance the FeP ratio and help the cells. In this case the activation of the pho regulon and e.g. better ROS resistance would be just a correlation but not the reason for the phenotype. The lack of the improvement in ΔphoB would then be caused by problems to adapt to -P specific issues. I am by no means an expert in P homeostasis or metabolism and this is speculation, but I could think of thermodynamic or kinetic problems in ATP dependent processes, the proton gradient or the mentioned NADPH/NADP+ ratios at relatively too high phosphate levels, specifically in cells which need to reduce the photosynthesis apparatus due to the iron limitation.
4. In order to prove that the phoB based regulation is the reason for the comparable better growth at -Fe-P, independent of the P concentration and the Fe to P ratio, it needs to be shown that activation of the pho regulon also at -Fe+P conditions improves the growth. At the very least an increased expression of sodB in -Fe+P should show an effect.

Minor points:
The paper is about Fe P ratios. In this context it should be mentioned in the paragraph "Phosphorus availability modulates sod expression in the world's oceans." that only SodB is iron dependent and that the other investigated SODs are Ni^{2+} (sodN), Mn^{2+} (sodA) and Zn^{2+} (sodC) dependent.
Line 94: FACS analysis is Fig S3 instead of S2
Figure 1: Can you also show absorption spectra of cells
Where all measurements in 1 B-E done after 4 days of growth? At what time were the cells

harvested for ROS measurements

Reviewer #3 (Remarks to the Author):

The present work by Qui and colleagues establishes PhoB as an important transcriptional regulator for mediating adaption to co-limitation of iron and phosphorus. The putative regulon of PhoB is proposed and selected targets are verified by various independent methods. Moreover, metagenomic and transcriptomic analysis shows the relevance gained in Synechocystis for marine cyanobacteria. The conducted experiments and the conclusions drawn are sound and logically. The legibility of the text is very good. Several comments:
Figure S2: The conclusions drawn from the immunoblot are hard to follow. Could you please also show a loading control and a semiquantitative analysis of the signals? I cannot follow the claim that PSI protein level is "strongly reduced", especially given that the Coomassie staining indicates that the loading is not fully equal.
Line 101 on which grounds did you pick these genes?
Line 105-106 I cannot follow the conclusion that P deficiency enhances SOD activity. The only thing shown is increased gene expression and enhanced activity in gel assays that are not normalized for SOD but total protein content, meaning that the increased sodB expression (which most likely leads to increased protein abundance) could be the sole reason for enhanced activity and not the enzyme activity per se.
Line 160-161 Fig 5 E and Fig S8 do not show growth patterns so I would rather call it a resembling response to -Fe+P cells.
For clarity, I would suggest to add the Fe/P conditions to Fig 5 E
How did the authors come up with the slr1908 gene as a potential phoB target?
Line 189 I think it is a bit far fetched to call pictures of flasks a physiological analysis. What about growth rate, O2 evolution, chlorophyll content? Looking at these cultures with the unaided eye seems like the flag tagged strains have a slight growth defect.
Line 193 please rephrase "target genes were enriched in genes"
Line 255 "globally important primary producers whose cell sizes are already close to"
It would add great value to the manuscript if the authors would mutate the most conserved residues to determine the relevant bases for Pho binding in gel shift assays.
Im not sure I missed it but why does sodB and slr1908 not show up in the chip seq results?
Why did the authors decide to use higher PhoB concentrations for gel-shift assays with petC1-petA?
For conclusions and introduction it would be great to mention that phoB is part of a two-component system and to state what is known about how this two-component system senses environmental stimuli.

Point-by-point Responses

Reviewer #1 (Remarks to the Author):

This work deals with the interplay between regulatory mechanisms relating responses to deficiency of various elements required for growth, namely Fe and P, in cyanobacteria. This condition is frequently found in natural environments. The authors point to the interest of the knowledge of mechanism relating P and Fe limitation in oceanic planktonic cyanobacteria; however, the present study has been performed on the model strain *Synechocystis* sp. PCC 6803, whose biology considerably differ from that of marine strains. It would have been interesting to study the mechanisms of adaptation to P/Fe limitation in *Prochorococcus* or marine *Synechococcus*. The data presented in Fig. 3 are rather indirect for this purpose. An important issue: the work presented needs improved (or clarified) statistic treatment. At first glance, some of the differences taken into account look of low statistical significance being the results of statistic tests rather unclear (see below).

RE: Thanks for your valuable comments and suggestions. In the revised manuscript, we have performed statistical analysis as you suggested. We agree that it would add great value if the present study were carried out in *Prochorococcus* or *Synechococcus*. However, genetic manipulations of marine cyanobacteria are not well established, particularly in the *Prochorococcus* strains. That was the reason we studied the adaptive strategy in a model strain and then expanded our findings to marine species.

-Page 4, for comparison of growth under different conditions, an estimation of growth rate constant (based on total protein or chlorophyll determination, or even OD) would be more adequate than cell number (also for data on Fig. 6D and 7A). Statistic comparison of data on photosynthetic activity and cell diameter presented in Fig. 1 is not clear. How many cells from how many cultures have been used for data in Fig. 1E? What is the p value corresponding to a, b and c in Figs. 1D and 1E? The same apply for Fig. 2A, 2B and 2D, Fig. 3A, Fig. 5C, 6C.

RE: Thanks for your comments. In the revised manuscript, we provided the growth rate of each strain according to your suggestion (Fig. 1d, Fig. 6d, and Fig. 7a), and the corresponding growth curve was presented in the supplementary files (Supplementary Fig. 13). According to your suggestion, the statistical analysis of the data was added to the results. In each experiment, the statistical significance across all treatments was calculated by one-way ANOVA, followed by Tukey's honest significant difference to test for significant differences among the means of each group. In cases involving comparison between two groups, we used a two-sided Student's *t*-test. Corresponding *P*-values were directly labeled in the figures or provided in the Source data file. Moreover, we also tried to be more precise in the description of the sampling method and experiment procedure. Details about the cell diameter determination were provided in the figure legend (line 714-718).

-Fig. 4. Data on Fig. 4A and 4B (in the absence and presence of MV) appears similar; thus, the sentence on lines 131-133 might not be sustained. Also, these results question the relationship between ETR and MV and superoxide levels (lines 138-140). (Again, statistical comparisons would be very informative here.)

RE: Thanks for your suggestions. To clarify the question you concerned, the original Fig. 4A and 4B were reorganized in the revised manuscript (Fig. 4a), and statistical analysis was added to show the difference between the treatments. The results clearly show that the ETR of Fe-limited samples

was less inhibited by MV when P deficiency occurred simultaneously.

-The role of PhoB on the regulation of sodB. In the phoB mutant, the expression of sodB in the absence of P is diminished with regard to the WT; however, in the mutant the levels are still higher in the absence than in the presence of P. Thus, PhoB could act as an enhancer of sodB expression, but other factor(s) would participate in the response to Fe/P availability. Please, comment on this. Please, label the different flasks in Fig. 5E.
RE: We agree with the idea that PhoB acts as an enhancer of *sodB* expression, but PhoB is not the only transcript factor that regulates *sodB* expression. In our Y1H library screening, the ROS responsive regulator RpaB was also found to interact with the *sodB* promoter (Supplementary Fig. 9). This regulator may contribute to the residual *sodB* induction in the *phoB* mutant. We added this information to the revised manuscript (line 163-166). Compared to RpaB, PhoB-mediated induction of target genes was more specific to the environmental P concentrations (line 243-245). This was the reason why we focused on the function of PhoB in Fe/P co-limitation acclimation. The corresponding flasks in Fig. 5e have been labeled in the revised version.

-The first paragraphs of the Discussion section deal with the connections of cellular adaptations under Fe and P limitations, including a higher requirement for Fe under conditions of P limitation (line 232-233) and the phenomenon of increased Fe content under P deficiency (lines 239-240). According to this, it would be expected that growth was higher under conditions of -P/+Fe than of -P/-Fe. It would be nice to include data on the -P/+Fe condition in the growth experiment of Fig. 1A.
RE: We included these data in Fig. 1a as suggested. The results show that growth of *Synechocystis* in the −P+Fe treatment was faster than in the −P−Fe treatment, as expected.

-Lines 288-289. Please change "universal role" to "widespread role".
RE: Thanks! Changed according to the suggestion (line 341-342).

Reviewer #2 (Remarks to the Author):

Qiu et al., show that the cyanobacterium *Synechocytis* PCC6803 grows better at combined Fe/P limitation than at Fe repletion alone. Furthermore, the authors show that the effect is lost in a ΔphoB strain. The PhoB regulon was investigated using ChIP-seq. Specifically sodB, slr1908 coding for an iron-selective porin and several genes involved in cyclic electron flow were identified to be directly positively regulated by PhoB. Finally, the authors propose that the improved growth at double depletion conditions is caused by maintaining intracellular ROS homeostasis, by enhancing Fe uptake capacity and by increasing cyclic electron flow.
These are interesting results that confirm the counterintuitive findings in other studies. I do not doubt the general effect and the data regarding the PhoB regulon, but I am not yet convinced by the interpretation of the results in terms of the -Fe+P and -Fe-P phenotypes. Several aspects remain puzzling to me. Those should be addressed prior to publication in a high-ranking journal.
Major concerns:
1. Despite being driven by previous data and the ChIP-seq results, the focus on sodB, slr1908 and

the CES genes as main contributors to the observed phenotype seems too narrow to me. I would rather recommend transcriptome analysis at -Fe and -P vs +P+Fe alone to cover the baseline transcriptional response at these conditions in order to compare it with the -Fe-P response to cover all transcriptional adaptions. In the current approach important changes might be missed.

RE: Thanks for your comments. Transcriptomic data are now provided (Fig. 7c and Supplementary Data 2). They show the whole picture of the transcriptional responses of *Synechocystis* under different Fe and P availabilities. We also analyzed the data as suggested, to identify genes that specifically respond to −Fe−P (Supplementary Data 3). Most of these genes encode proteins with unknown functions. The genes with known functions include the chaperonin gene (*clpB*), a gene that controls carbon metabolism (*slr6041*), etc. The contribution of these genes in Fe/P co-limitation acclimation will be further assessed in the future.

This study aims to reveal how P deficiency enhances the growth and fitness of Fe-limited cyanobacteria. Generally, maintaining ROS homeostasis and ensuring sufficient Fe uptake are the main challenges of Fe-limited cyanobacteria. The synergistic regulation of PhoB in the expression of *sodB* and Fe uptake gene *slr1908* led us to focus on the roles of PhoB in Fe/P co-limitation acclimation. In the revised manuscript, we extended our views to other PhoB-dependent or independent pathways that may regulate Fe/P co-limitation (e.g., line 271-277, line 317-321, etc.).

2. The general iron stress response by Fur/Isar1 is completely neglected in the data interpretation and discussion.

a. The majority of the transcripts that are discussed to cause the improved growth at -Fe-P (sodB, petF, Cytb6f) are actively repressed at -Fe through IsaR1. Actually, sodB is the textbook example of a gene that is repressed at iron limitation in a wide range of organism (E. coli, S aureus, Shewanella ...) including PCC6803. The ferrodoxin (petF) is e.g. replaced by a copper dependent alternative plastocyanin (petE) to spare iron. This indicates that it is in general a fitness advantage to repress these genes under iron limitation. It is not obvious to me why this regulation should have survived evolution if, in fact, it is the reason for a fitness disadvantage.

b. All iron transporters should be strongly up-regulated by the Fur regulon at -Fe. I would assume that the cells already max their Fe uptake capacity at iron limitation alone. Slr1908 might not be Fur dependent, but transcriptome data from PCC6803 show that it is induced at -Fe and -P conditions in a similar strength (Kopf et al 2014). Also, the cited paper (Qiu et al 2021) states that "the expression of slr1908 was significantly upregulated in Fe-deplete medium". Even if the levels are further increased at -Fe-P in addition to all the other iron up-take systems, it needs to be shown that this actually increases the intracellular iron concentrations. Slr1908 seems to be a major iron transporter and even be relevant at iron replete conditions: " slr1908 is highly expressed also under Fe-replete conditions. When grown in iron-sufficient medium, the slr1908KD strain exhibits a range of phenotypes that, taken together, are clearly indicative of iron limitation: lowered intracellular Fe quotas, induction of isiA and lowered PSI content and activity" (Qiu 2021). Thus, it seems not

surprising to me that the slr1908kd does very bad at -Fe and can not be recovered by -P.

RE: Thank you for your comments. We included in the introduction a description of the current understanding of the Fur repressor (line 68-71) and have integrated the regulatory role of IsaR1 in our model for interpretation (line 262-263).

In the revised manuscript, we systematically strengthened the reliability of the data on PhoB-mediated gene regulation. Although the interaction between PhoB and *petC1-petA* promoter was supported by our experimental data, the genes encoding the NDH complex failed to be identified as PhoB targets (Supplementary Data 1). Thus, we removed the content related to the CET pathways (petF, Cytb6f) from the revised version. However, the regulatory role of PhoB on *sodB* expression was further strengthened in the revised manuscript by using the PhoB-activated strain $PhoR^{T412N}$ and showing that *sodB* was induced in the $PhoR^{T412N}$ strain (Supplementary Fig. 17).

In our opinion, repressing the genes encoding Fe-containing proteins under iron limitation should be a fitness advantage, which helps to save Fe from non-essential processes for essential processes in Fe-limited cyanobacteria. However, non-essential doesn't mean not important. Overexpression of *sodB* can greatly reduce the intracellular ROS content of Fe-limited cyanobacteria (Supplementary Fig. 11), and the importance of its induction was also discussed in lines 257-261. But *sodB* induction is only one of the reasons for the enhanced fitness under −Fe−P; the growth rate of SodB-OE is even lower than the WT, implying that concerted mechanisms including the induction of Fe uptake genes are involved in the improved growth under −Fe−P vs −Fe+P (line 262-286).

To further compare the expression level of *slr1908* under −Fe+P and −Fe−P conditions, we introduced an 8*His tag into the native *slr1908* locus. Western blotting with monoclonal antibodies showed that the Slr1908 protein is indeed more highly expressed under −Fe−P than −Fe+P (Supplementary Fig. 12). Although both Kopf et al and our previous data showed that *slr1908* is induced under −Fe conditions, this phenomenon was not observed in our Western blot assay (Supplementary Fig. 12), transcriptomic data (Supplementary Data 2), and qRT-PCR (Fig. 6c). This is probably due to the difference in sampling time. Hernández-Prieto et al (2012) showed that *slr1908* upregulates 2-fold at the first 12 h of Fe-limitation but gradually decreases to +Fe level after 3 days of growth. Thus, under our experimental conditions in the present study, *slr1908* is mainly induced by P deficiency.

We carried out the suggested experiment and found that Fe content under −Fe−P was indeed increased compared with that under −Fe+P (Supplementary Fig. 18). And this phenomenon, i.e. P deficiency increases intracellular Fe content, was even more remarkable under Fe sufficient conditions. We also agree that *slr1908*KD already does very badly at −Fe and it is not surprising that it cannot be recovered by −P. What surprised us is that this strain grew even worse under −Fe−P than under −Fe+P condition, indicating *slr1908* function is more important under −Fe−P than −Fe+P (line 194-196).

Thank you very much for your suggestions. Hopefully our data interpretations in the revised

manuscript are satisfactory.

3. Together the above points leave me with two possible explanations:
a. If the interpretation of the authors is correct, the iron stress regulation of PCC6803 is sub-optimal at -Fe+P conditions. PhoB could lead to a re-allocation of the sparse iron from other iron dependent proteins to CES and sodB. That could be potentially seen in transcriptome data. However, are -Fe+P conditions so rare in natural environments that there is no selective pressure to optimize the iron stress regulation during evolution? Is there anything known of the Fe and P concentrations at diverse environments and seasons?
b. In an alternative potential explanation, the organisms do the best possible adaption to the -Fe+P conditions and the high P levels are for some reason detrimental by themself if the cells are iron depleted. In this interpretation the cells are not able to keep the optimal ratios (e.g. Redfield ratio) at -Fe+P and hence the lowered phosphate levels alone rebalance the FeP ratio and help the cells. In this case the activation of the pho regulon and e.g. better ROS resistance would be just a correlation but not the reason for the phenotype. The lack of the improvement in ΔphoB would then be caused by problems to adapt to -P specific issues. I am by no means an expert in P homeostasis or metabolism and this is speculation, but I could think of thermodynamic or kinetic problems in ATP dependent processes, the proton gradient or the mentioned NADPH/NADP+ ratios at relatively too high phosphate levels, specifically in cells which need to reduce the photosynthesis apparatus due to the iron limitation.

RE: We believe that the regulation in response to iron limitation in PCC6803 is suboptimal under −Fe+P conditions. Those organisms adapted better to −Fe−P than −Fe+P have been widely reported in *Crocosphaera* (Yang et al. 2022. *ISME J*, **16**: 2702–2711), *Trichodesmium* (Walworth et al. 2016. *Nat Commun*, **7**: 12081), and different types of land plants (*Nam* et al. 2021. *Nat Commun*, **12**: 7211). Moreover, Therby-Vale et al (2022. *Trends Plant Sci*, **27**: 502–509) stated that iron deficiency-induced chlorosis can be reverted by the depletion of other micronutrients (i.e., Mn) or macronutrients (i.e., S or P), and the modulation of antioxidant homeostasis could be an important mechanism.

To our knowledge, the well-defined Fe/P co-limited regions include large areas of the western North Pacific and North Atlantic Ocean. However, its presence may be more pervasive than expected. We also anticipate that the geographic extent of these regions could vary seasonally, and over even longer time scales, depending on ocean atmosphere interactions (upwelling and turbulent mixing). It has been reported that the starvation response in *E. coli* is activated when the extracellular Pi concentration is approximately 4 μM. This value exceeds the highest Pi concentration found anywhere in the open ocean, including Pi-enriched deep-sea habitats (Karl. D.M. 2014, *Ann Rev Mar Sci.* **6:** 279-337). From this aspect, full repression of Pho regulon in natural environments is almost impossible. The response of cyanobacteria to low Fe habitats should be a combined effect of Fur and PhoB regulators. Moreover, we believe that when enhanced Fe uptake capacity induced by

−P cannot fulfil the additional Fe demand, cyanobacteria under −Fe−P would cut off the cellular Fe costs to *sodB* and respond as they did under −Fe+P.

In the revised manuscript, we tried to be more precise in making the conclusions (line 202-204), and the functions of differentially expressed genes that are not regulated by PhoB were also discussed (line 253-255 and line 271-277).

4. In order to prove that the phoB based regulation is the reason for the comparable better growth at -Fe-P, independent of the P concentration and the Fe to P ratio, it needs to be shown that activation of the pho regulon also at -Fe+P conditions improves the growth. At the very least an increased expression of sodB in -Fe+P should show an effect.

RE: Thanks for your suggestions. We carried out the following experiments and found that:

Overexpression of SodB significantly decreased the intracellular ROS content of Fe-limited cyanobacteria but resulted in a further decline in the growth rate (Supplementary Fig. 11).

Overexpression of Slr1908 enhanced the growth rate of Fe-limited cyanobacteria (Supplementary Fig. 11), and suppression of Slr1908 expression (*slr1908KD*) resulted in serious growth defect of co-limited cyanobacteria (Fig. 6d).

Both *sodB* and *slr1908* were significantly induced in a Pho regulon-activated strain $PhoR^{T412N}$ (Supplementary Fig. 17), and their induction in PhoB mutant was significantly decreased, demonstrating the essential role of PhoB in the up-regulation of these genes.

The Pho regulon-activated strain $PhoR^{T412N}$ exhibited a significantly decreased growth rate under −Fe+P. This is probably due to over-allocated energy and resources into useless processes (e.g. massively synthesis of alkaline phosphatase, PstCSAB, etc.) under stressful conditions.

Overall, our results indicate that PhoB plays an essential role in the growth and fitness of co-limited cyanobacteria.

Minor points:

The paper is about Fe P ratios. In this context it should be mentioned in the paragraph "Phosphorus availability modulates sod expression in the world's oceans." that only SodB is iron dependent and that the other investigated SODs are Ni2+ (sodN), Mn2+ (sodA) and Zn2+ (sodC) dependent.

RE: Thanks for your suggestion. The relevant information has been provided in the revised manuscript (line 119-121).

Line 94: FACS analysis is Fig S3 instead of S2

RE: The corresponding information has been corrected (line 98).

Figure 1: Can you also show absorption spectra of cells

RE: The absorption spectra of each treatment have been provided in Fig. 1e.

Where all measurements in 1 B-E done after 4 days of growth? At what time were the cells harvested for ROS measurements

RE: We now provide a more detailed description of the sampling method and experiment procedure in the Figure legend. Statistical analysis of the data was also systematically improved.

Reviewer #3 (Remarks to the Author):

The present work by Qui and colleagues establishes PhoB as an important transcriptional regulator for mediating adaption to co-limitation of iron and phosphorus. The putative regulon of PhoB is proposed and selected targets are verified by various independent methods. Moreover, metagenomic and transcriptomic analysis shows the relevance gained in Synechocystis for marine cyanobacteria. The conducted experiments and the conclusions drawn are sound and logically. The legibility of the text is very good. Several comments:

Figure S2: The conclusions drawn from the immunoblot are hard to follow. Could you please also show a loading control and a semiquantitative analysis of the signals? I cannot follow the claim that PSI protein level is “strongly reduced”, especially given that the Coomassie staining indicates that the loading is not fully equal.

RE: Thanks for your suggestions. Loading controls, such as β-actin and tubulin are absent from cyanobacteria, instead, Coomassie blue-stained gels were used as the control (e.g., Edgar et al. 2012. *Nature*, **485**: 459–464). To ensure that the proteins were loaded in an equal amount, in the revised manuscript, Coomassie blue-stained gels were quantified by densitometry, and a semiquantitative analysis was provided according to your suggestion (Supplementary Fig. 2 and Supplementary Fig. 12). The description of the data was also improved in the revised version (line 92-96).

Line 101 on which grounds did you pick these genes?

RE: The reason we picked these genes was explained in line 105-106.

Line 105-106 I cannot follow the conclusion that P deficiency enhances SOD activity. The only thing shown is increased gene expression and enhanced activity in gel assays that are not normalized for SOD but total protein content, meaning that the increased sodB expression (which most likely leads to increased protein abundance) could be the sole reason for enhanced activity and not the enzyme activity per se.

RE: Thank you. We have rephrased the wording in the revised manuscript (line 110-111).

Line 160-161 Fig 5 E and Fig S8 do not show growth patterns so I would rather call it a resembling response to -Fe+P cells.

RE: Thanks for your suggestion. The corresponding sentence has been changed in the revised version (line 171).

For clarity, I would suggest to add the Fe/P conditions to Fig 5 E

RE: The corresponding flasks in Fig. 5e have been labeled in the revised version.

How did the authors come up with the slr1908 gene as a potential phoB target?

RE: In the revised manuscript, we improved the logical connection between each part, and the reason we picked *slr1908* as a potential PhoB target has been provided in line 180-182.

Line 189 I think it is a bit far fetched to call pictures of flasks a physiological analysis. What about growth rate, O_2 evolution, chlorophyll content? Looking at these cultures with the unaided eye seems like the flag tagged strains have a slight growth defect.

RE: We provided the relevant information in the revised manuscript as you suggested (Supplementary Fig. 8). The flag-tagged strains indeed have a slightly decreased chlorophyll content, despite their photosynthetic activity resembling that of the WT. Furthermore, in the updated version, we used a combination of motif scanning and transcriptomic analysis to identify the PhoB targets (line 202-218). And the PhoB targets discussed in our study were systematically tested in a Pho regulon-activated strain (line 274-277). ChIP-seq results were only utilized to check the reliability of our computational analysis (line 216-218).

Line 193 please rephrase "target genes were enriched in genes"

RE: Thanks, we have rephrased this sentence in the revised version (line 219-221).

Line 255 "globally important primary producers whose cell sizes are already close to"

RE: The corresponding sentence has been corrected (line 295-296).

It would add great value to the manuscript if the authors would mutate the most conserved residues to determine the relevant bases for Pho binding in gel shift assays.

RE: We carried out the suggested experiment. Phylogenetic analysis shows that the TTAA motif was highly conserved (Supplementary Fig. 7c). Mutations in any of these three TTAA sequences in *slr1908* promoter remarkably reduced or abolished the DNA binding ability of PhoB (Supplementary Fig. 7d), indicating that they are vital for recognition by PhoB (line 207-212). This feature was further utilized to identify the putative Pho regulons (line 212-218). Thank you very much for your valuable suggestion.

Im not sure I missed it but why does sodB and slr1908 not show up in the chip seq results?

RE: The Chip-seq results for *sodB* and *slr1908* are shown in Supplementary Fig. 14 in the revised manuscript.

Why did the authors decide to use higher PhoB concentrations for gel-shift assays with petC1-petA?

RE: We increased PhoB concentration because the retarded band of petC1-petA promoter was somewhat diffused. In fact, the retarded band of petC1-petA was also visible when PhoB concentration was similar to that of other EMSA experiments. In the revised manuscript, the discussion about petC1-petA and the cyclic electron transport pathway has been removed from the main text due to the lack of the PhoB binding site in the promoter regions of NDH genes.

For conclusions and introduction it would be great to mention that phoB is part of a two-component system and to state what is known about how this two-component system senses environmental stimuli.

RE: Thanks for your suggestion. The relevant information has been provided in the first paragraph of the discussion section (line 243-245).

Reviewer #1 (Remarks to the Author):

This work deals with the interplay between regulatory mechanisms relating responses to deficiency of various elements required for growth, namely Fe and P, in cyanobacteria. This condition is frequently found in natural environments, and thus the study is of high interest. In this new version of the manuscript, the authors have satisfactorily addressed my concerns raised on the first version, including data analysis and statistical treatment. Thus, in this version the methodology is sound and the conclussion supported by the results obtained.

Reviewer #2 (Remarks to the Author):

In their revision, the authors could address my previous concerns.

I have the following additional comments.

Most data are from Synechocystis, this should be more reflected in title and abstract.

It would be helpful if the authors try to define some nutrient level scenarios at which both the Pho and the Fur are of relevance, and when additional Pho regulation is not helpful anymore. It could be discussed that many "iron stress studies" use an chelator to remove all biologically available iron, while in this study iron was "only" omitted from the medium, which should leave iron traces and iron storage of the cells less affected.

Given the observed interconnection of the iron (Fur) and phosphate (PhoR) regulatory circuits it might be also of interest that the function of both regulators is dependent on FtsH in Synechocystis, which might represent act as an epistatic factor.

Reviewer #3 (Remarks to the Author):

The authors have addressed everything I criticized and it was a pleasure to read the revised manuscript, the authors have added some data for a nice story, only two minor things:

"Loading controls, such as β-actin and tubulin are absent from
cyanobacteria, instead, Coomassie blue-stained gels were used as the control (e.g., Edgar et al. 2012.
Nature, 485: 459–464). To ensure that the proteins were loaded in an equal amount, in the revised
manuscript, Coomassie blue-stained gels were quantified by densitometry, and a semiquantitative analysis was provided according to your suggestion (Supplementary Fig. 2 and Supplementary Fig. 12). The description of the data was also improved in the revised version (line 92-96)"

Reply: There are several housekeeping proteins in Synechocystis that can be detected by commercially available antibodies (i.e. RbcR or TrxA). Im a bit confused now, did you perform a densitometric analysis of the stained gel as you claim in the figure caption or of the signals on the membrane? I think you have clearly shown that PSI protein is reduced when comparing -Fe-P and -Fe+P treatment as for these two lanes the protein amount in the gel looks similar, however you state that "Samples were loaded on an equal total protein basis" and looking at these coomassie gels this is clearly not the case when you only load half and a quarter for the +Fe+P treatment (I suppose thats what the numbers mean?).

Line 213 and following:
You use the term "regulon" in a wrong way here as you refer to the different potential target genes of PhoB when you state "92 regulons were upregulated". The regulon however is the set of target genes of a given regulator in its entirety. Please correct.

Point-by-point Responses

We thank all reviewers for their positive comments and feedback. We have addressed their concerns point by point below in blue.

Point-by-point Responses

Reviewer #1 (Remarks to the Author):
This work deals with the interplay between regulatory mechanisms relating responses to deficiency of various elements required for growth, namely Fe and P, in cyanobacteria. This condition is frequently found in natural environments, and thus the study is of high interest. In this new version of the manuscript, the authors have satisfactorily addressed my concerns raised on the first version, including data analysis and statistical treatment. Thus, in this version the methodology is sound and the conclussion supported by the results obtained.
RE: Thanks for your valuable suggestions and positive feedback.

Reviewer #2 (Remarks to the Author):
In their revision, the authors could address my previous concerns.
I have the following additional comments.
-Most data are from Synechocystis, this should be more reflected in title and abstract.
RE: Thanks for your suggestions. Changed as requested.

-It would be helpful if the authors try to define some nutrient level scenarios at which both the Pho and the Fur are of relevance, and when additional Pho regulation is not helpful anymore. It could be discussed that many "iron stress studies" use an chelator to remove all biologically available iron, while in this study iron was "only" omitted from the medium, which should leave iron traces and iron storage of the cells less affected.
RE: Thanks for your valuable suggestions. We discussed relevant information as you suggested (line 330-335 and 361-362).

-Given the observed interconnection of the iron (Fur) and phosphate (PhoR) regulatory circuits it might be also of interest that the function of both regulators is dependent on FtsH in Synechocystis, which might represent act as an epistatic factor.
RE: Thanks. The revised manuscript has discussed relevant information in line 312-315.

Reviewer #3 (Remarks to the Author):
The authors have addressed everything I criticized and it was a pleasure to read the revised manuscript, the authors have added some data for a nice story, only two minor things:

-"Loading controls, such as β-actin and tubulin are absent from cyanobacteria, instead, Coomassie blue-stained gels were used as the control (e.g., Edgar et al. 2012. Nature, 485: 459–464). To ensure that the proteins were loaded in an equal amount, in the revised manuscript,

Coomassie blue-stained gels were quantified by densitometry, and a semiquantitative analysis was provided according to your suggestion (Supplementary Fig. 2 and Supplementary Fig. 12). The description of the data was also improved in the revised version (line 92-96)"

Reply: There are several housekeeping proteins in Synechocystis that can be detected by commercially available antibodies (i.e. RbcR or TrxA). Im a bit confused now, did you perform a densitometric analysis of the stained gel as you claim in the figure caption or of the signals on the membrane? I think you have clearly shown that PSI protein is reduced when comparing -Fe-P and -Fe+P treatment as for these two lanes the protein amount in the gel looks similar, however you state that "Samples were loaded on an equal total protein basis" and looking at these coomassie gels this is clearly not the case when you only load half and a quarter for the +Fe+P treatment (I suppose thats what the numbers mean?).

RE: Thanks for your valuable suggestions. We did perform a densitometric analysis of the stained gel before the Western blot assay. The corresponding description has been added to the figure legend.

-Line 213 and following:

You use the term "regulon" in a wrong way here as you refer to the different potential target genes of PhoB when you state "92 regulons were upregulated". The regulon however is the set of target genes of a given regulator in its entirety. Please correct.

RE: Thank you. Corrected as suggested.
